# S100A9 is a Biliary Protein Marker of Disease Activity in Primary Sclerosing Cholangitis

**DOI:** 10.1371/journal.pone.0029821

**Published:** 2012-01-11

**Authors:** Lisa Reinhard, Christian Rupp, Hans-Dieter Riedel, Thomas Ruppert, Thomas Giese, Christa Flechtenmacher, Karl Heinz Weiss, Petra Kloeters-Plachky, Wolfgang Stremmel, Peter Schirmacher, Peter Sauer, Daniel Nils Gotthardt

**Affiliations:** 1 Department of Internal Medicine IV, University Hospital of Heidelberg, Heidelberg, Germany; 2 Deptartment of Proteomics, Center for Molecular Biology, University of Heidelberg, Heidelberg, Germany; 3 Department of Immunology, University Hospital of Heidelberg, Germany; 4 Department of Pathology, University Hospital of Heidelberg, Germany; Carl-Gustav Carus Technical University-Dresden, Germany

## Abstract

**Background and Aims:**

Bile analysis has the potential to serve as a surrogate marker for inflammatory and neoplastic disorders of the biliary epithelium and may provide insight into biliary pathophysiology and possible diagnostic markers. We aimed to identify biliary protein markers of patients with primary sclerosing cholangitis (PSC) by a proteomic approach.

**Methods:**

Bile duct-derived bile samples were collected from PSC patients (n = 45) or patients with choledocholithiasis (n = 24, the control group). Liquid chromatography-tandem mass spectrometry (LC-MS/MS) was performed to analyse the proteins, 2-D-gel patterns were compared by densitometry, and brush cytology specimens were analysed by RT-PCR.

**Results:**

A reference bile-duct bile proteome was established in the control group without signs of inflammation or maligancy comprising a total of 379 non-redundant biliary proteins; 21% were of unknown function and 24% had been previously described in serum. In PSC patients, the biliary S100A9 expression was elevated 95-fold (p<0.005), serum protein expression was decreased, and pancreatic enzyme expression was unchanged compared to controls. The S100A9 expression was 2-fold higher in PSC patients with high disease activity than in those with low activity (p<0.05). The brush cytology specimens from the PSC patients with high disease activity showed marked inflammatory activity and leukocyte infiltration compared to the patients with low activity, which correlated with S100A9 mRNA expression (p<0.05).

**Conclusions:**

The bile-duct bile proteome is complex and its analysis might enhance the understanding of cholestatic liver disease. Biliary S100A9 levels may be a useful marker for PSC activity, and its implication in inflammation and carcinogenesis warrants further investigation.

## Introduction

Cholestatic liver disease (CLD) is a major cause of liver damage and failure. CLD pathogenesis ranges from choledocholithiasis, which is often completely relieved by endoscopic intervention, to chronic diseases such as primary sclerosing cholangitis (PSC), which may require liver transplantation [1], [2], [3] [4]. For some CLD pathologies (e.g., transporter defects), the underlying pathological mechanisms have been thoroughly elucidated, whereas for others, including PSC and post-transplant cholangiopathies, the pathophysiological basis of the disease and disease progression remain much less clear [5]. Some forms of CLD are complicated by malignancy, specifically cholangiocarcinomas. Cholangiocarcinomas are associated with a poor prognosis, in part due to being detected at later stages when resection is no longer feasible [1], [6]. Recent advances in endoscopic retrograde cholangiography (ERC) and its wide-spread use in treating CLD have allowed for sampling bile duct-derived bile during routine examinations [7].

Bile is secreted by hepatocytes into the canaliculi and is then continuously modified by cholangiocytes. The major constituents of bile include bile acids (BA) and phospholipids, which are primarily composed of phosphatidylcholine, and cholesterol. The lipid components of bile are complemented by proteins, predominantly mucins [8], [9]. Despite bile composition research having the potential to improve the understanding of PSC pathogenesis and enhance the development of new cholangiocarcinoma biomarkers, little is known about the protein components of bile. Due to interference between bile contents and proteomic analysis methods, a comprehensive proteomic analysis of bile duct-derived bile samples is technically more challenging than similar comprehensive analyses of other biological fluids, such as plasma, urine, and liquor. Few publications have reported data on biliary protein profiles, but a recent study that compared peptide patterns using a special mass spectrometry approach demonstrated expression differences in patients with cholestatic liver disease [10].

In this study, we performed a comprehensive analysis of the bile duct-derived bile proteome to identify the differences in biliary protein expression profiles among patients with CLD. We specifically focused on PSC, with the goals of gaining new insights into CLD pathophysiology and establishing a strong basis for the discovery of new diagnostic markers.

## Materials and Methods

### Patients and Bile Sampling during ERC

Bile-duct sampling was performed in patients undergoing ERC prior to any therapeutic procedures and after selective intubation of the papilla. Bile samples were mixed with protease inhibitors (Complete, EDTA-free, Roche, Germany), snap frozen in liquid nitrogen, and stored at −80°C until further use. Bile samples from 69 patients were included in this study. 24 of the patients had been diagnosed with choledocholithiasis, and 45 patients had PSC. Diagnosis of PSC was made based on typical ERC findings, and all the PSC patients received ursodeoxycholic acid. The clinical data are presented in Table 1. None of the patients had a history of variceal bleeding. The Mayo risk score (MRS) was calculated for the PSC patients [11], and the endoscopic disease activity in them was classified as follows: 1) high disease activity: ≥3 balloon dilatations/year, ≥2 episodes of clinical biliary obstruction (i.e., jaundice, abdominal pain), or progression of biliary tree destruction as determined by the ERC, and 2) low disease activity: all other PSC patients. Samples from patients with apparent bacterial and/or fungal biliary infections were excluded. All procedures in this study were compliant with the Declaration of Helsinki and were approved by a local ethics committee (Ethikkommission der Medizinischen Fakultät Heidelberg, Alte Glockengießerei 11/1 D-69115 Heidelberg). All patients provided written informed consent.

**Table 1:** Patient characteristics.

[median ± SEM]	Control	PSC	PSC low activity	PSC high activity	PSC low MRS	PSC interm. MRS
N	24	45	26	19	25	20
F/M	9/15	11/34	7/19	4/15	6/19	5/15
Age [yrs]	50±3.7	42±1.8	40±2.3	50±2.9	38±2.0	45±3.1
Bilirubin [mg/dl]	1.1±0.5	1.0±0.3	0.9±0.08	2.0±0.5	0.8±0.08	1.8±0.5
C-reactive protein	8.4±16.2	4.2±6.7	2.0±0.6	11.6±13.8	2.0±0.6	11.6±13.8
WBC [/nl]	7.0±0.9	6.2±0.4	6.9±0.3	5.0±0.8	6.2±0.3	6.9±0.8
AP [IU/l]	167±74	229±31	178±32	293±57	174±33	284±56
GGT [IU/l]	415±132	112±54	109±49	153±106	115±52	89±104
AST [IU/l]	61±139	46±5	33±4	64±10	33±4	63±10
ALT [IU/l]	191±66	44±10	40±10	47±19	44±11	45±18
Albumin	42.3±1.8	41.0±0.8	43.8±0.7	39.7±1.4	44.0±0.6	39.3±1.3
Mayo Risk Score	N/A	−0.16±0.14	−0.34±0.09	0.90±0.19	−0.38±0.07	0.81±0.16

MRS Mayo risk score; PSC low and high activities refer to the endoscopic finding.

### Sample Preparation, 2D Gel Electrophoresis, and mass spectrometry

Excess albumin and IgG were removed from bile samples. First, 200 µl of bile was centrifuged for 10 min at 13,000 g to remove debris. Depending on the protein concentration, 25–100 µl of resin-coupled antibodies against albumin and IgG (Albumin and IgG Removal Kit, Amersham Biosciences) was added after washing the resin with PBS, and incubated for 60′. The resin was removed by centrifugation. To remove lipophilic substances, the following precipitation protocol was established based on a protocol for plant protein extraction [12]: 1 ml of 0.1 M ammonium acetate in 100% methanol was added to 200 µl of bile at −20°C, vortexed and incubated overnight. The precipitated protein was pelleted at 13,000 g, the supernatant was discarded, the pellet was washed twice with 1.2 ml 0.8 M ammonium acetate/80% methanol at −20°C, and then the pellet was washed twice with 80% acetone. The resulting pellet was air dried on ice. Protein concentration was measures by 2-D-Quant kit (Amersham Biosciences). For the 2-D SDS-PAGE, the pellets were resuspended in lysis buffer containing 7 M urea, 2 M thiourea (Merck), 2% (w/v) CHAPS, 1% (w/v) DTT (Sigma), 2% (v/v) Pharmalyte 3–10 (Amersham Biosciences), and protease inhibitor mixture. The extracts were separated in the first dimension using 18-cm strips with immobilised non-linear gradients ranging from pH 3 to 11 (Amersham Biosciences) and in the second dimension using a standard SDS-PAGE technique [13], [14]. The 2-D gels were stained with either silver or colloidal Coomassie dye. The procedures were performed under standardised conditions for all the gels. Total BA concentrations were determined enzymatically using 3alpha- hydroxysteroid dehydrogenase (Enzabile; Nycomed, Oslo, Norway).

Following SDS-PAGE separation and Coomassie staining, the proteins were analysed by mass spectrometry. Detailed information on the parameters can be found in the supplemental material.

### 1-D Gel Electrophoresis and total S100 measurement

The protein samples were separated by 1-D SDS-PAGE (Nu-Page 4–12% Bis-Tris-Gel, Invitrogen Life Technologies, USA) and stained with either silver or colloidal Coomassie dye. The total S-100 protein levels were measured using a routine electrochemiluminescence assay (ECLIA; Elecsys S-100® assay; Roche® Diagnostics; Mannheim, Germany).

### Cytopathological evaluation and expression analyses

Brush cytology specimens were obtained during ERC in cases of unclear strictures. All the biliary brushing specimens were initially fixed in 95% ethyl alcohol, and different stainings were performed for cytological evaluation: PAP (Papanicolau), HE (hematoxylin and eosin), and PAS (periodic acid-Schiff). The diagnoses were made by the pathologist. The remaining brushes were immediately placed in tubes containing RNAlater (Qiagen, Hilden, Germany). The S-100A9 mRNA expression levels were analysed using standard kits (Search-LC, Heidelberg, Germany). The gene expression was normalised to that of the housekeeping gene cyclophilin B.

### Image Processing, Dataset, and Statistical Analyses

Digitised images of 2-D gels from the series were analysed using the ImageJ Software (Rasband, W.S., ImageJ, US National Institutes of Health, Bethesda, Maryland, USA, http://rsb.info.nih.gov/ij/, 1997–2009) for spot detection, gel matching, and background correction, and these data were normalised to the sum of the total spot volume.

Data on protein and gene names, predicted molecular weights, and pI values were retrieved from UniProt (www.expasy.ch) [15]. Pathways proteins are involved in were analysed by using UniProt and Genecards databases (www.expasy.ch; www.genecards.com).

All statistical analyses were performed using the PASW 17 software (SPSS Inc., Chicago, Illinois). Non-parametric Mann-Whitney U test was used for continuous data, and Fisher's exact test was used where applicable. All p values <0.05 were considered to be statistically significant.

## Results

### Establishment and improvement of a biliary protein purification protocol

**Figure 1:**
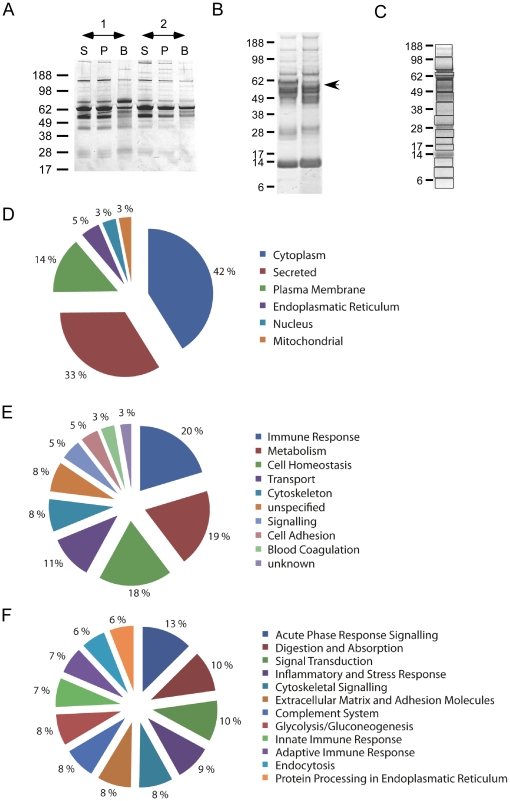
Bile purification and identification of biliary proteins. (**A**) Comparison of serum (S), plasma (P), and bile duct-derived bile proteins (B) from two choledocholithiasis patients demonstrates many similar expressed proteins; however, the protein composition differs between the serum and bile samples. (**B**) Albumin and IgG depletion and purification for the crude bile duct-derived bile samples. The purification of the bile samples was monitored using 1-D silver stained gels, and the crude bile samples (Lane 1) were compared to the precipitated samples depleted of albumin and IgG. The arrow indicates the marked reduction in albumin. (**C**) A mixture of bile samples was separated by 1-D Gel electrophoresis, cut into the depicted slices and analysed by mass spectrometry. The 301 identified proteins with a Mascot score >100 were classified according to reported subcellular localisation (**D**), the biological processes in which they are involved (**E**) and the pathways the majority of proteins are involved (**F**).

The bile protein expression profiles in the 1-D SDS-PAGE separation from the patients with choledocholithiasis were similar to the serum and plasma profiles from the same patients (Fig. 1A). Furthermore, the bile samples were found to contain varying concentrations of albumin, which was the most predominant bile protein in most of the analysed samples. As high concentrations of albumin strongly interfere with measurements by masking the proteins in lower abundance and by limiting the success of isoelectric focusing (IEF), we removed the excess albumin and IgG from the bile samples. Using this approach, >80% of the albumin was removed from each sample (Fig. 1B). The silver-stained gels of crude bile and purified bile (albumin removal plus protein precipitation) were compared. The total protein loss following precipitation alone was less than 20%, as determined by the control experiments (data not shown). Albumin was the only protein removed in significant amounts; otherwise, the overall protein pattern was unchanged following the purification (Fig. 1B).

### A comprehensive analysis of the bile duct bile proteome

**Figure 2:**
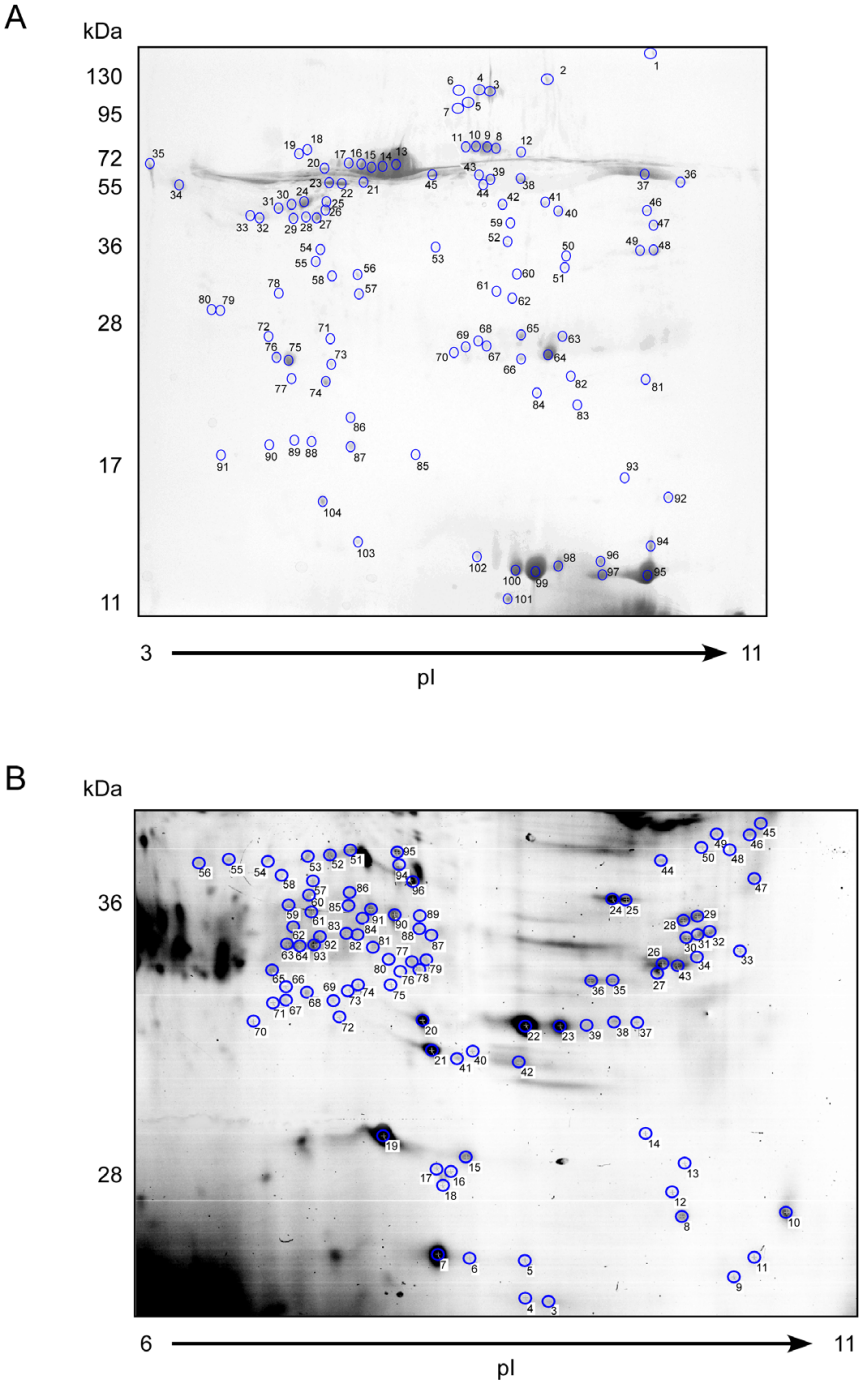
2-D SDS-PAGE analysis of purified bile. 2-D gels of biliary proteins. pH range of the first-dimension isoelectric focusing gel is shown at the bottom, and the sizes of the molecular mass standards (in kDa) for the second dimension are indicated on the left. The proteins corresponding to the spots on the gels (circled in blue) were identified by mass spectrometry. (**A**) 500 µg of protein was separated on a gradient from pH 3–11 (non-linear). To identify less abundant proteins, (**B**), 1 mg of protein was separated; more prominent spots were excluded, and the less prominent spots were identified by mass spectrometry.

Bile samples from four choledocholithiasis patients who showed no signs of cholangitis nor malignancy (i.e., an absence of clinical and laboratory signs) or other biliary tract abnormalities were analysed. A total of 1,500 µg of bile proteins (375 µg from each of the four samples) were mixed and purified as outlined in the Materials and Methods section. A total of 500 µg or 1 mg of the purified proteins was separated using 2-D SDS-PAGE and stained with colloidal Coomassie dye (Fig. 2A,B). All the visible protein spots were selected and analysed by MS-MS Maldi-TOF mass spectrometry. To identify all the proteins that were not separated in the IEF step, a second sample of 50 µg of purified protein was separated by 1-D SDS-PAGE only and the proteins were determined by mass spectrometry (Fig. 1C). By combining these methods, we were able to unambiguously identify 379 proteins with a high Mascot score. Of the 379 identified proteins, 301 were characterised in the Uniprot Knowledgebase (Table S1), and 78 were hypothetical, predicted, or otherwise not further defined (Table S2). Furthermore, we identified 558 proteins with a Mascot score <100 and/or with only one uniquely identified peptide. These proteins were considered to be identified with less certainty and were therefore separated and included in the Supplemental Material (Table S2, S3). Of these proteins, the most prominent and abundant proteins on the 2-D gel were primarily found in the plasma/serum in addition to the bile. By comparison, 130 of the 301 known proteins had been previously identified in proteomic studies of the serum and plasma. We classified the bile proteins according to the biological processes they have been identified as performing and by their molecular function, predicted subcellular localisation, and tissue expression. The majority of the bile proteins were predicted to be cytosolic (124/301), secreted (100/301), or plasma membrane-associated (42/301). Bile proteins predicted to be associated with the nucleus or mitochondria were uncommon (Fig. 1D). The biological processes associated with the primarily identified known proteins included immune response (62/301), metabolism (57/301), and cell homeostasis (57/301). For 23 of the 301 characterised proteins, the function was unknown or could not be clearly classified (Fig. 1E). The most frequent pathways proteins are involved in were Acute Phase Signaling (21/301), Digestion and Absorption (17/301), Signal Transduction (17/301), Inflammatory and Stress Response (15/301) and Cytoskeletal Signaling (14/301). (Fig. 1F). In total 40 pathway could be identified (Table S4). For 19 proteins the pathway they are involved in could not be identified.

### Biliary protein levels are associated with disease activity in patients with PSC

**Figure 3:**
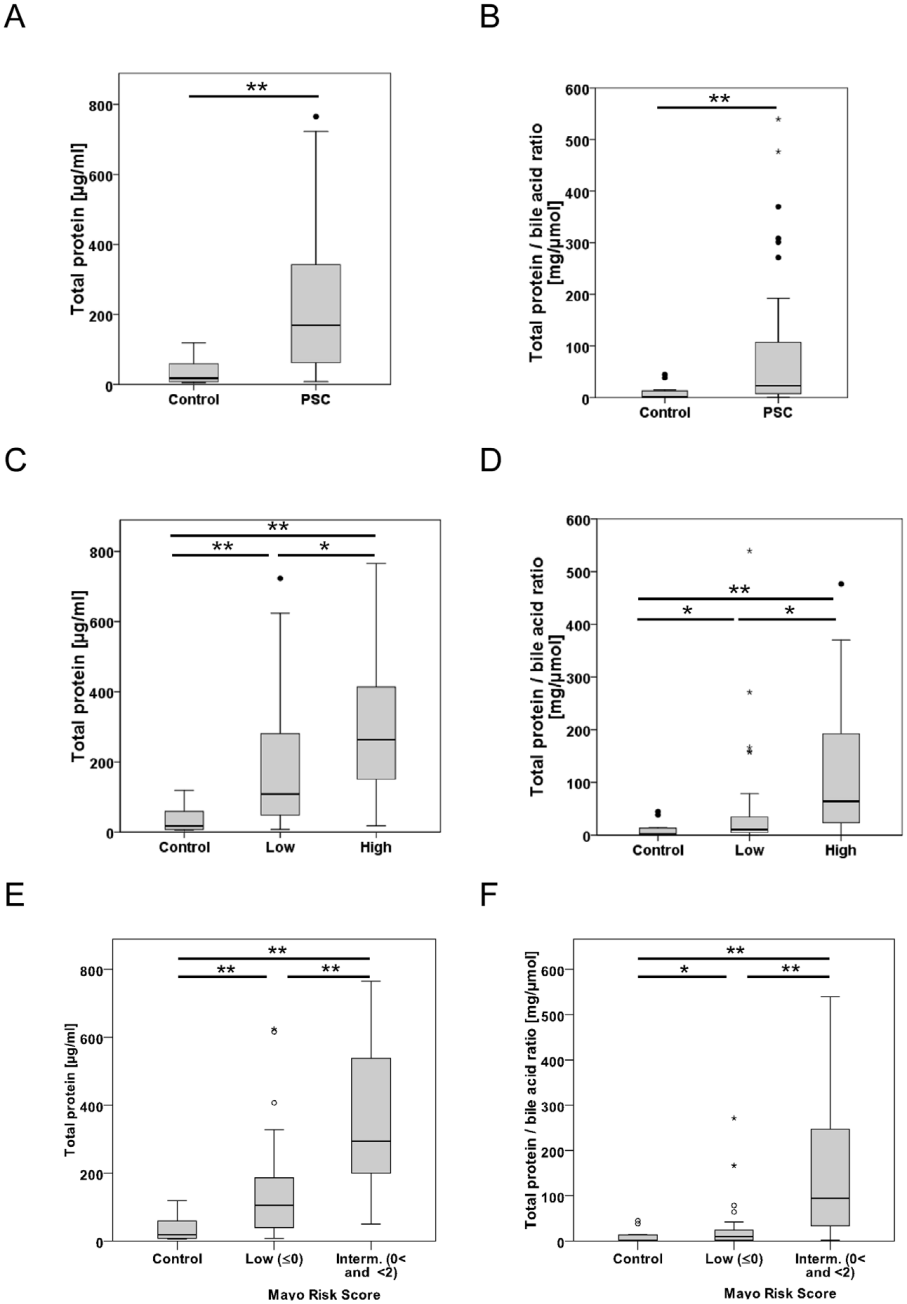
Correlation between biliary proteins and disease activity in PSC patients. Bile from PSC patients showed a marked increase in biliary protein content. (**A**) Box plots of the protein concentration per volume show a marked increase for the PSC patients. If the PSC patients were classified based on disease activity, either by endoscopic findings (**C**) or by Mayo Risk score (**E**), the corresponding biliary protein concentrations showed significantly greater concentrations in the patients with high PSC activity than in those with low activity. If the bile samples were normalised by the bile-acid content, the results were similar (**B,** controls versus all the PSC patients; **D,** PSC patients classified by endoscopic findings; **F,** PSC patients classified by Mayo Risk Score). Box plots of the protein concentration normalised by the BA concentration. (control = 24, PSC = 45, low MRS = 25, intermediate MRS = 20; low endoscopic = 26; high endoscopy activity = 19; ns = not significant; *p<0.05; **p<0.005).

Bile from the PSC patients (n = 46) showed markedly elevated biliary protein concentrations compared to controls (n = 24) (272±295 mg/µl vs. 118±410 mg/µl, respectively; p<0.001; Fig. 3A), which was even more pronounced when normalised by the bile-acid concentration (86±131 mg/µmol vs. 66±288 mg/µmol, respectively; p<0.001; Fig. 3C). A comparison between the PSC patients based on their endoscopic disease activity showed that the biliary-protein content and the protein content normalised by the BA concentration were both significantly elevated in the high-disease-activity PSC patient group (n = 19; 372±366 mg/µl and 124±143 mg/µmol, respectively) compared to the low-activity group (n = 27; 201±213 mg/µl and 59±117 mg/µmol, respectively; p<0.05). Both of the groups had greater total biliary-protein content and biliary-protein content normalised by the BA concentration compared to the controls (Fig. 3B,D). If the PSC patients were categorised by their Mayo risk scores (MRS), the total and normalised protein contents were significantly different between the low-MRS and intermediate-MRS patients (Fig. 3E,F).

### The comparison of the protein expression profiles identified marked upregulation of S100A9

**Figure 4:**
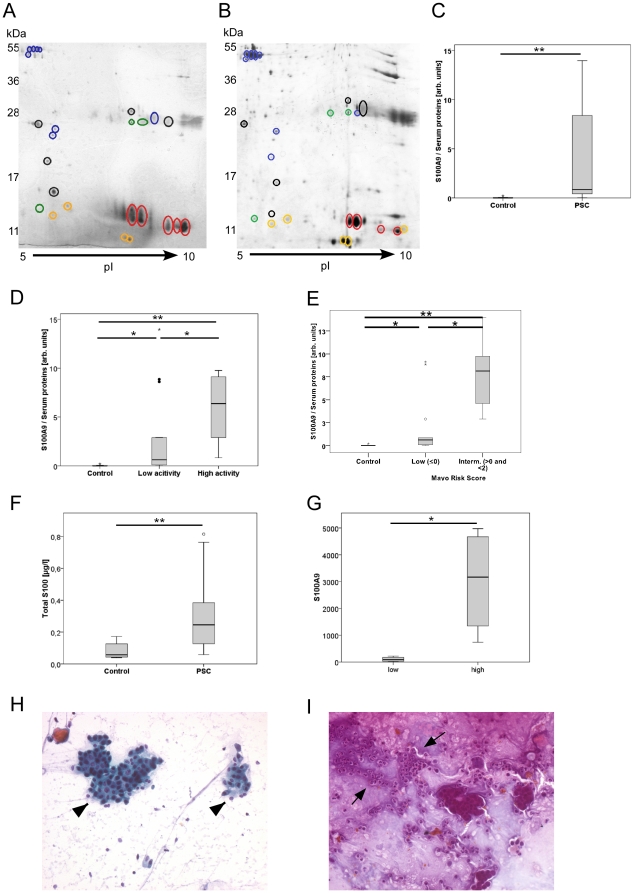
Identification of S100A9 as a marker of disease activity. The protein patterns among the PSC and choledocholithiasis (control) patients were compared. Intensities of 25 spots uniquely identified by mass spectrometry were measured using densitometry. The spot resembling S100A9 was markedly upregulated in the PSC patients with high activity (**B**) compared to those with low activity (**A**). Circled spots: haemoglobin (red), S100A9 (yellow), serum proteins (black), pancreatic enzymes (green), and others (blue). An evaluation of the relative amount of S100A9 in the spots demonstrated a clear upregulation of S100A9 in the PSC patients (**C**). Subgroup analyses of the PSC patients performed according to disease activity revealed a distinct increase in S100A9 in the PSC patients with high disease activity relative to those with low disease activity (**D**). The PSC patients were also stratified by their Mayo risk scores. (n: control = 6; PSC = 18; low MRS = 13; intermediate MRS = 5; low endoscopic = 12; high endoscopic = 6). The total S100 levels were significantly elevated in the PSC patients PSC (**F**) (*p<0.05; **p<0.005). The RT-PCR analyses of the brush cytology samples demonstrated marked upregulation of S100A9 expression (n = 8, 4 in the low activity group and 4 in the high activity group; p<0.05) (**G**).The brush cytology from the patients with unclear strictures demonstrated corresponding cytological findings. The cytology from a patient with low disease activity showed regular epithelial cells (arrowheads) (**H**), whereas the cytology from a patient with high disease activity showed leukocyte infiltrate (arrows) and altered epithelial cells (**I**).

To identify disease-dependent alterations in the relative amounts of specific proteins, a subset of the protein spots on the 2-D gel was quantified using densitometry and normalised to protein content, as outlined above (Fig. 4A,B). The expression levels of S100A9 were significantly increased in the PSC patients (Fig. 4C). The S100A9 expression was elevated by approximately 95-fold relative to the baseline serum proteins (0.04±0.09 arbitrary units vs. 3.83±4.43 arbitrary units, respectively; p<0.005; n = 6 controls and n = 19 PSC patients). When comparing the control patients to the patients with low- or high-activity PSC, the S100A9 expression was elevated 67-fold and 137-fold, respectively (0.04±0.09 vs. 2.88±4.56 and 0.04±0.09 vs. 5.89±3.64, respectively; p<0.05 and p<0.005, respectively; n = 6 controls, n = 13 low-activity PSC patients, and n = 6 high-activity PSC patients). The S100A9 expression was increased 2-fold in patients with high disease activity (p<0.05) (Fig. 4D). When the Mayo risk scores were compared, S100A9 was significantly different between the patients with low and intermediate MRS (Fig. 4E). A comparison of the pancreatic enzymes chymotrypsinogen B and triacylglycerol lipase showed no significant differences between the control and PSC patients or between the two PSC subgroups. We validated these results in 37 samples from additional patients (n = 8 controls and n = 29 PSC patients with low disease activity) by measuring the total S100 protein concentration in aspirated bile. In these samples, the total S100 levels were 0.31±0.23 µg/l in the PSC group and 0.08±0.05 µg/l in the control group (p<0.001; Fig. 4F).

### S100A9 expression analyses in brush cytology samples

The S100A9 mRNA expression levels were also analysed in the brush cytology samples from the PSC patients with unclear stenosis. The S100A9 mRNA expression was found to be upregulated by approximately 30-fold in the brush cytology samples from the PSC patients with high endoscopic disease activity relative to those with low disease activity (3,012±1998 vs. 102±95, respectively; p<0.05; n = 8; Fig. 4G).

The cytological analyses of the brush samples from the PSC patients with low and high disease activities displayed inflammatory cell infiltration and inflammatory alterations of epithelial, which correlated with the S100A9 levels detected in the corresponding bile samples (Fig. 4H,I)

## Discussion

In this study, we identified nearly 400 proteins in bile duct-derived bile of patients without inflammation and malignancy. Furthermore, we demonstrated that bile protein expression profiles are disease-dependent in PSC patients and that profile alterations reflect changes at the epithelial level. Therefore, as a complement to the significant data available on the lipid composition of bile, the biliary protein expression profile uncovered here provides new mechanistic insights into cholestatic disease. These insights may have the potential to lead to new therapeutic targets.

Bile-sample protein analysis is hampered by the overall protein concentration in bile being low, and the low molecular weight compounds, such as BAs, phospholipids, and bilirubin, are present in high concentrations and strongly interfere with IEF, gel electrophoresis, and further biochemical analyses. Protein constitutes approximately 5% of the dry weight of bile [16] and is derived from serum, hepatocytes, and bile duct epithelial cells [17].

Using our purification method, we were able to identify the components of the bile duct-derived bile proteome. Previous studies focused on analysis of gallbladder bile or bile from patients with hepatobiliary malignancy [18], [19], [20], [21], [22] [23], [24]. In both cases, these analyses would result in a bile proteome significantly different from that of bile duct-derived bile in a non-malignant setting. The physiological bile duct-derived bile proteome established in the current study may serve as a standard of reference for further studies and complements the data of the previous studies. In addition, we identified 78 bile proteins whose functions are not yet known. Proteins with a Mascot score of less than 100, which are presented in the Supplemental Material section, accounted for an additional 467 candidate bile proteins and will be the focus of future studies. To assure specificity, we included only proteins with a Mascot score greater than or equal to 100 and with a minimum of two unique peptides.

The precise origin of individual bile proteins is unclear. We detected only minor concentrations of cellular components from the mitochondria, nucleus, and cytoskeleton. These findings provide evidence that biliary proteins in bile duct-derived bile are not cellular debris. Many of the proteins in bile, including albumin, immunoglobulins, haptoglobin, haemoglobin subunits, transferrin, fibrinogen, and components of the complement system, are also found in human serum; their concentrations in bile are approximately 0.2% of their serum concentrations [25]. Other bodily fluids, such as urine [26] and tears [27], also contain serum proteins at minor concentrations relative to blood. Different factors are responsible for the presence of serum proteins in bile fluid. Due to the concentration gradient between the serum and bile, serum proteins may pass through the tight junctions between hepatocytes and biliary epithelial cells and enter the bile fluid [28]. Furthermore, large amounts of albumin, and possibly other serum proteins, are secreted directly into bile by hepatocytes [29]. Transcytosis may contribute to the movement of proteins in bile, and receptor-mediated protein endocytosis at the hepatocyte sinusoidal membrane has been shown for IgA in rats [30]. Furthermore, the exocytosis of lysosomal enzymes and co-transport of phospholipids have also been demonstrated for bile [31]. Interestingly, the biliary protein content in PSC patients was found to be associated with chronic inflammation and demonstrated a positive correlation with the disease activity. This finding was highlighted when the patients were categorised by their endoscopic findings and by established Mayo risk score stratifications.

The S100A9 protein was originally discovered as an immunological protein in neutrophils and has more recently been associated with several forms of human cancer and inflammation-associated carcinogenesis. When combined with S100A8, S100A9 forms the heterodimeric protein calprotectin (S100A8/9), which is expressed in nearly all the cells, tissues, and fluids of the human body [32]. S100A8/9 appears to exhibit antimicrobial and antiproliferative activities and may regulate inflammation, as increased S100A8/9 concentrations were observed during the acute-phase reaction. The S100A8/9 heterodimer is most likely secreted by monocytes and granulocytes, and its extracellular effects are mediated through the RAGE (receptor for advanced glycation end products), a multiligand receptor of the immunoglobulin family [32].

In the bile from the PSC patients, the S100A9 concentration increased with disease activity and was significantly greater in the PSC patients relative to the control patients with choledocholithiasis. Increased S100A9 levels have also been associated with a variety of other inflammatory diseases. S100A8/9 overexpression was first discovered in cystic fibrosis, but later studies have also demonstrated elevated S100A8/9 levels in active chronic inflammatory bowel disease and in the synovial fluid of patients with rheumatoid arthritis [32]. A potential positive role for S100A8/9 in dendritic cells has also been proposed in the context of primary biliary cirrhosis pathogenesis [33]. Even though S100A8/9 is not specific for a particular disease entity and its levels fluctuate considerably between individual patients, it may nevertheless help in identifying inflammatory processes in patients in the absence of diagnostic clinical findings. Furthermore, recently published clinical and experimental data suggest an important role for changes in the expression and/or function of S100 proteins in carcinogenesis [32], [34].

Bile has the potential to serve as a surrogate substance for further analysis; therefore, we performed experiments to characterise bile and confirm its components at the cellular level. Bile analysis is an attractive option for diseases confined to the biliary epithelium and/or diseases that represent a non-homogeneous distribution pattern in the liver. These include PSC and (early) cholangiocarcinoma, as cells involved in these diseases are often not easily accessed. As outlined above, therefore, bile is an attractive integrative parameter for identifying either diagnostic markers or immunologically-active substances. To determine whether the changes in the bile-protein profiles were also exhibited by the epithelial cells, we performed an analysis of the brush cytology samples acquired during ERC. We found that both the cytological analyses and quantitative real-time PCR analysis of the S100A9 mRNA expression levels confirmed our results from the 2-D gel comparison of the bile-protein samples. This result suggests that our approach is generally feasible and that further studies (e.g., determining changes in differentiation between PSC and cholangiocarcinoma) are promising. Furthermore, it will be interesting to correlate these findings with data from the ongoing genome-wide association studies of PSC patients and in the context of bicarbonate-mediated protection of the biliary epithelium [35]–[36].

In conclusion, in the current study, we defined a proteome of bile derived from the bile duct in a non-inflammatory and non-malignant setting that could serve as a reference point for further studies. Furthermore, we identified the biliary S100A9 level as a marker for disease activity in PSC patients and confirmed these findings at the cellular level by brush cytology samples. Therefore, biliary sampling and the analysis of bile markers should be tested as a routine diagnostic tool for patients with cholestatic liver diseases.

## Supporting Information

Table S1 **List of bile duct-derived bile proteins identified by mass spectrometry including additional information on predicted protein characteristics.**
(DOCX)

Table S2 **List of predicted bile duct-derived bile proteins separated by a Mascot score >100 or <100.**
(DOCX)

Table S3 **List of bile duct-derived bile proteins identified with a Mascot score <100.**
(DOCX)

Table S4 **List of pathways to which duct-derived bile proteins belong.**
(DOCX)
