# Supplementary material for: S100A9 is a Biliary Protein Marker of Disease Activity in Primary Sclerosing Cholangitis
Source: PLoS One. 2012 Jan 11;7(1):e29821. doi: 10.1371/journal.pone.0029821 (PMC3256182; doi:10.1371/journal.pone.0029821)
Supplement: Table S1 — List of bile duct-derived bile proteins identified by mass spectrometry including additional information on predicted protein characteristics. (DOCX) [file pone.0029821.s001.docx]

**Supplemental Table 1**

List of bile duct-derived bile proteins identified by mass spectrometry including additional information on predicted protein characteristics

| **No.** | **Score^1^** | **Protein name** | **Biological Process** | **Molecular Function** | **Predicted Location** | **Expressed** | **Gene name** | **MW kDa (predicted)** | **pI (predicted)** | **Serum^2^** | **LC MS/MS** | **Spot No Gel A** | **Spot No Gel B** |
| --- | --- | --- | --- | --- | --- | --- | --- | --- | --- | --- | --- | --- | --- |
| **1** | 1 | Alpha-1B-glycoprotein | Unknown | Unknown | Secreted | Liver | A1BG | 54 | 5.6 | + | + | 18,19 |  |
| **2** | 1 | Alpha-2-macroglobulin | Immune Response | Enzyme Regulator | Secreted | Liver | A2M | 165 | 6.0 | + | + |  |  |
| **3** | 2 | ATP-binding cassette sub-family B member 4 | Transport | Binding | Plasma membrane | Liver | ABCB4 | 142 | 8.7 | - | + |  |  |
| **4** | 3 | ATP-binding cassette sub-family B member 5 | Transport | Binding | Plasma membrane | Others | ABCB5 | 90 | 8.4 | - | + |  |  |
| **5** | 3 | ATP-binding cassette sub-family B member 11 | Transport | Transporter | Plasma membrane | Hepatocytes | ABCB11 | 146 | 6.2 | - | + |  |  |
| **6** | 2 | Short-chain specific acyl-CoA dehydrogenase, mitochondrial | Metabolism | Enzyme | Mitochondrial | Widely expressed | ACADS | 44 | 8.1 | - | + |  |  |
| **7** | 1 | Actin alpha 1 | Cytoskeleton | Protein Binding | Cytoplasm | Others | ACTA1 | 42 | 5.2 | - | + |  |  |
| **8** | 1 | Actin, cytoplasmic 1 | Cytoskeleton | Protein Binding | Cytoplasm | Widely expressed | ACTB | 42 | 5.3 | + | + |  |  |
| **9** | 1 | Alpha actinin 4 | Cytoskeleton | Protein Binding | Cytoplasm | Widely expressed | ACTN4 | 105 | 5.3 | - | + |  |  |
| **10** | 2 | Beta-centractin | Cytoskeleton | Binding | Cytoplasm | Widely expressed | ACTR1B | 42 | 6.0 | - |  | 43 |  |
| **11** | 3 | Alcohol dehydrogenase 1B | Metabolism | Enzyme | Cytoplasm | Liver | ADH1B | 40 | 8.6 | + | + |  |  |
| **12** | 1 | afamin precursor (Transport protein) | Transport | Protein Binding | Secreted | Liver | AFM | 69 | 5.6 | + | + |  |  |
| **13** | 1 | Angiotensinogen | Signalling | Receptor Activity | Secreted | Liver | AGT | 53 | 5.9 | + | + |  |  |
| **14** | 2 | Alpha-2-HS-glycoprotein | Immune Response | Binding | Secreted | Liver | AHSG | 39 | 5.4 | + | + |  |  |
| **15** | 2 | Adenylate kinase 1 | Cell Homeostasis | Enzyme | Cytoplasm | Ubiquitous | AK1 | 22 | 8.7 | - | + |  |  |
| **16** | 1 | Alcohol dehydrogenase | Metabolism | Enzyme | Cytoplasm | Ubiquitous | AKR1A1 | 37 | 6.3 | - | + |  |  |
| **17** | 1 | Aldo-keto reductase family 1 member C1 | Metabolism | Enzyme | Cytoplasm | Liver | AKR1C1 | 37 | 8.0 | - | + |  |  |
| **18** | 3 | Aldo-keto reductase family 1 member C2 | Metabolism | Enzyme | Cytoplasm | Liver | AKR1C2 | 37 | 7.1 | - | + |  |  |
| **19** | 3 | Aldo-keto reductase family 1 member C3 | Metabolism | Enzyme | Cytoplasm | Widely expressed | AKR1C3 | 48 | 9.5 | - | + |  |  |
| **20** | 1 | Aldo-keto reductase family 1 member C4 | Metabolism | Enzyme | Cytoplasm | Liver | AKR1C4 | 37 | 6.7 | - |  |  |  |
| **21** | 1 | Albumin | Transport | Protein Binding | Secreted | Liver | ALB | 71 | 6.1 | + | + | 13-17,21, 25,28,44,45,67-69, 79,93-95, 104 | 7 |
| **22** | 1 | Retinal dehydrogenase 1 | Metabolism | Enzyme | Cytoplasm | Liver | ALDH1A1 | 55 | 6.3 | - | + |  |  |
| **23** | 3 | 10-formyltetrahydrofolate dehydrogenase | Metabolism | Enzyme | Cytoplasm | Liver | ALDH1L1 | 99 | 5.6 | - | + |  |  |
| **24** | 1 | 4-trimethylaminobutyraldehyde dehydrogenase | Metabolism | Enzyme | Cytoplasm | Liver | ALDH9A1 | 53 | 5.1 | - | + |  |  |
| **25** | 1 | Fructose-bisphosphate aldolase A | Metabolism | Enzyme | Cytoplasm | Ubiquitous | ALDOA | 39 | 8.3 | + | + |  |  |
| **26** | 2 | Alkaline phosphatase, tissue-nonspecific isozyme | Metabolism | Enzyme | Plasma membrane | Widely expressed | ALPL | 41 | 5.6 | + | + |  |  |
| **27** | 3 | Protein AMBP | Immune Response | Enzyme Regulator | Secreted | Liver | AMBP | 43 | 5.6 | + | + |  |  |
| **28** | 1 | pancreatic amylase alpha 2A | Metabolism | Enzyme | Secreted | Pancreas | AMY2A | 58 | 6.6 | - | + |  |  |
| **29** | 1 | pancreatic amylase alpha 2B | Metabolism | Enzyme | Secreted | Pancreas | AMY2B | 58 | 6.6 | + | + |  |  |
| **30** | 1 | Alanine aminopeptidase, membrane | unspecified | Enzyme | Plasma membrane | Widely expressed | ANPEP | 110 | 5.3 | + | + |  |  |
| **31** | 1 | Annexin A13 | unspecified | Binding | Plasma membrane | Other GI | ANX13 | 35 | 5.5 | - | + |  |  |
| **32** | 3 | Annexin A11 | Cytoskeleton | Protein Binding | Cytoplasm | Widely expressed | ANXA11 | 54 | 7.5 | - | + |  |  |
| **33** | 3 | Annexin A2 | Cell Adhesion | Protein Binding | Secreted | Widely expressed | ANXA2 | 39 | 7.6 | - | + |  |  |
| **34** | 2 | Annexin A4 | Cell Homeostasis | Binding | Cytoplasm | Epithelium | ANXA4 | 36 | 5.8 | - | + |  |  |
| **35** | 1 | Annexin A5 | Blood coagulation | Binding | Secreted | Widely expressed | ANXA5 | 36 | 4.9 | - |  | 56,57,91 |  |
| **36** | 1 | Serum amyloid P-component | Immune Response | Binding | Secreted | Liver | APCS | 25 | 6.1 | + | + |  |  |
| **37** | 3 | DNA-(apurinic or apyrimidinic site) lyase | Cell Homeostasis | Enzyme | Nucleus | Widely expressed | APEX1 | 36 | 8.3 | - |  |  |  |
| **38** | 1 | Apolipoprotein A-I | Transport | Binding | Secreted | Liver | APOA1 | 31 | 5.6 | + |  | 75,76 | 24 |
| **39** | 1 | Apolipoprotein A-IV | Transport | Binding | Secreted | Other GI | APOA4 | 45 | 5.3 | + | + | 30,31 |  |
| **40** | 1 | Apolipoprotein B-100 | Transport | Binding | Secreted | Liver | APOB | 516 | 6.1 | + | + |  |  |
| **41** | 2 | Apolipoprotein D | Transport | Binding | Secreted | Liver | APOD | 213 | 5.1 | - | + |  |  |
| **42** | 1 | Apolipoprotein E | Transport | Binding | Secreted | Widely expressed | APOE | 36 | 5.7 | + |  | 58 |  |
| **43** | 1 | Beta-2-glycoprotein 1 | Blood coagulation | Binding | Secreted | Liver | APOH | 38 | 8.3 | + | + |  |  |
| **44** | 3 | Adenine phosphoribosyltransferase | Cell Homeostasis | Enzyme | Cytoplasm | Ubiquitous | APRT | 20 | 5.8 | - | + |  |  |
| **45** | 2 | ADP-ribosylation factor-related protein 1 | Transport | Enzyme | ER | Widely expressed | ARFRP1 | 23 | 7.5 | - | + |  |  |
| **46** | 2 | Rho GDP-dissociation inhibitor 1 | unspecified | Receptor Activity | Cytoplasm | Ubiquitous | ARHGDIA | 23 | 5.0 | - |  | 72 | 35,36 |
| **47** | 1 | Actin-related protein 2/3 complex subunit 5-like protein | Cytoskeleton | Protein Binding | Cytoplasm | Widely expressed | ARPC5L | 17 | 6.2 | - |  | 85 |  |
| **48** | 1 | V-type proton ATPase catalytic subunit A | Cell Homeostasis | Enzyme | Cytoplasm | Ubiquitous | ATP6V1A | 68 | 5.4 | - | + |  |  |
| **49** | 2 | Attractin-2 | Immune Response | Receptor Activity | Secreted | Blood cells | ATRN | 158 | 7.2 | - | + |  |  |
| **50** | 2 | Zinc-alpha-2-glycoprotein | Metabolism | Unknown | Secreted | Widely expressed | AZGP1 | 37 | 5.6 | + | + |  |  |
| **51** | 3 | Beta-2-microglobulin | Immune Response | Protein Binding | Cytoplasm | Widely expressed | B2M | 14 | 6.1 | + | + | 43 |  |
| **52** | 1 | Spliceosome RNA helicase BAT1 | Cell Homeostasis | Enzyme | Nucleus | Ubiquitous | BAT1 | 30 | 5.5 | - | + |  |  |
| **53** | 1 | Biliverdin reductase A | Metabolism | Enzyme | Cytoplasm | Liver | BLVRA | 33 | 6.1 | - | + |  |  |
| **54** | 1 | Flavin reductase | Cell Homeostasis | Enzyme | Cytoplasm | Liver | BLVRB | 22 | 7.1 | + |  | 82 |  |
| **55** | 3 | Complement C1s subcomponent | Immune Response | Enzyme | Secreted | Liver | C1S | 77 | 4.9 | + | + |  |  |
| **56** | 1 | Complement C3 | Immune Response | others | Secreted | Liver | C3 | 187 | 6.0 | + |  | 2 |  |
| **57** | 1 | Complement component C4-A | Immune Response | others | Secreted | Liver | C4A | 193 | 6.7 | + | + | 62 |  |
| **58** | 2 | Complement C4-B | Immune Response | others | Secreted | Liver | C4B | 193 | 6.7 | + | + | 6,7 |  |
| **59** | 1 | C4b-binding protein alpha chain | Immune Response | Enzyme Regulator | Secreted | Liver | C4BPA | 67 | 7.2 | + | + |  |  |
| **60** | 2 | Complement component 9 | Immune Response | others | Secreted | Liver | C9 | 63 | 5.4 | + | + |  |  |
| **61** | 1 | Carbonic anhydrase 1 | Cell Homeostasis | Enzyme | Cytoplasm | Ubiquitous | CA1 | 29 | 6.6 | + | + | 65 |  |
| **62** | 1 | Carbonic anhydrase 2 | Cell Homeostasis | Enzyme | Cytoplasm | Ubiquitous | CA2 | 29 | 6.9 | + | + |  |  |
| **63** | 2 | Calmodulin | Signalling | Enzyme | Cytoplasm | Widely expressed | CALM1 | 17 | 4.1 | - |  |  | 10 |
| **64** | 3 | Calnexin | unspecified | Binding | ER | Widely expressed | CANX | 68 | 4.5 | - | + |  |  |
| **65** | 3 | Calpain 1 | unspecified | Enzyme | Cytoplasm | Ubiquitous | CAPN1 | 82 | 5.5 | - | + |  |  |
| **66** | 3 | F-actin-capping protein subunit beta | Cytoskeleton | Protein Binding | Cytoplasm | Widely expressed | CAPZB | 31 | 5.4 | - | + |  |  |
| **67** | 3 | Catalase | Metabolism | Enzyme | Cytoplasm | Ubiquitous | CAT | 60 | 6.9 | + | + |  |  |
| **68** | 3 | Carbonyl reductase 1 | Metabolism | Enzyme | Cytoplasm | Liver | CBR1 | 30 | 8.6 | - | + |  |  |
| **69** | 3 | CD5 antigen-like | Immune Response | Immune Response | Secreted | Spleen, lymph node | CD5L | 38 | 5.3 | - |  |  | 54 |
| **70** | 2 | Monocyte differentiation antigen CD14 | Immune Response | Receptor Activity | Plasma membrane | Blood cells | CD14 | 40 | 5.8 | + | + |  |  |
| **71** | 2 | Scavenger receptor cysteine-rich type 1 protein M130 | Immune Response | Protein Binding | Secreted | Blood cells/Liver | CD163 | 125 | 5.6 | + | + |  |  |
| **72** | 2 | Cadherin-1 | Cell Adhesion | Protein Binding | Plasma membrane | Epithelium | CDH1 | 97 | 4.6 | - | + |  |  |
| **73** | 1 | Cadherin-related family member 2 | Cell Adhesion | Binding | Plasma membrane | Liver | CDHR2 | 142 | 4.3 | - | + |  |  |
| **74** | 3 | Carcinoembryonic antigen-related cell adhesion molecule 1 | Cell Adhesion | Protein Binding | Plasma membrane | Epithelium | CEACAM1 | 58 | 5.7 | - | + |  |  |
| **75** | 3 | Carcinoembryonic antigen-related cell adhesion molecule 6 | Unknown | Unknown | Plasma membrane | Unknown | CEACAM6 | 63 | 4.0 | - | + |  |  |
| **76** | 2 | Chymotrypsin-like elastase family member 2A | Metabolism | Enzyme | Secreted | Pancreas | CELA2A | 29 | 8.8 | - | + |  |  |
| **77** | 3 | Chymotrypsin-like elastase family member 2B | Metabolism | Enzyme | Secreted | Pancreas | CELA2B | 29 | 6.5 | - | + |  |  |
| **78** | 2 | Chymotrypsin-like elastase family member 3A | Metabolism | Enzyme | Secreted | Pancreas | CELA3A | 29 | 6.4 | - | + |  |  |
| **79** | 3 | Chymotrypsin-like elastase family member 3B | Metabolism | Enzyme | Secreted | Pancreas | CELA3B | 29 | 5.7 | - | + |  |  |
| **80** | 1 | Liver carboxylesterase 1 | Metabolism | Enzyme | ER | Liver | CES1 | 62 | 6.2 | + | + |  |  |
| **81** | 1 | Complement factor B | Immune Response | others | Secreted | Liver | CFB | 86 | 6.7 | + | + |  |  |
| **82** | 1 | Complement factor H | Immune Response | others | Secreted | Liver | CFH | 139 | 6.2 | + | + |  |  |
| **83** | 3 | Complement factor H-related protein 1 | Immune Response | Enzyme Regulator | Secreted | Liver | CFHR1 | 38 | 7.4 | + | + |  |  |
| **84** | 1 | Complement factor I | Immune Response | Enzyme | Secreted | Liver | CFI | 66 | 7.7 | + | + |  |  |
| **85** | 2 | Charged multivesicular body protein 4b | Transport | Protein Binding | Cytoplasm | Widely expressed | CHMP4B | 25 | 4.8 | - | + |  |  |
| **86** | 3 | Similar to cytoskeleton-associated protein 4 | Unknown | Unknown | ER | Widely expressed | CKAP4 | 42 | 8.2 | - | + |  |  |
| **87** | 2 | Tetranectin | unspecified | Protein Binding | Secreted | Plasma | CLEC3B | 23 | 5.5 | + | + |  |  |
| **88** | 1 | Chloride intracellular channel protein 1 | Transport | others | Cytoplasm | Liver | CLIC1 | 27 | 5.1 | + | + |  |  |
| **89** | 1 | Clusterin (Apolipoprotein J) | Unknown | Protein Binding | Secreted | Widely expressed | CLU | 52 | 5.9 | + | + |  | 44 |
| **90** | 1 | UMP-CMP kinase | Cell Homeostasis | Enzyme | Cytoplasm | Ubiquitous | CMPK1 | 48 | 8.2 | - | + |  |  |
| **91** | 2 | Cytosolic non-specific dipeptidase | Metabolism | Enzyme | Cytoplasm | Liver | CNDP2 | 53 | 5.7 | - | + |  |  |
| **92** | 3 | Coronin-1A | Cytoskeleton | Protein Binding | Cytoplasm | Widely expressed | CORO1A | 51 | 6.3 | + | + |  |  |
| **93** | 1 | Ceroluplasmin | Transport | Binding | Secreted | Liver | CP | 122 | 5.4 | + | + |  | 17 |
| **94** | 2 | Pancreatic carboxypeptidase A1 | Metabolism | Enzyme | Secreted | Pancreas | CPA1 | 47 | 5.5 | + | + |  |  |
| **95** | 2 | Carboxypeptidase A2 | Metabolism | Enzyme | Secreted | Pancreas | CPA2 | 47 | 5.7 | - | + |  |  |
| **96** | 1 | Carboxypeptidase B | Metabolism | Enzyme | Secreted | Pancreas | CPB1 | 33 | 5.5 | - | + |  |  |
| **97** | 3 | Carboxypeptidase M | Metabolism | Enzyme | Plasma membrane | Widely expressed | CPM | 51 | 6.9 | - | + |  |  |
| **98** | 1 | Quinone oxidoreductase | Metabolism | Binding | Cytoplasm | Widely expressed | CRYZ | 35 | 8.6 | - | + |  |  |
| **99** | 3 | Cystatin SN | Signalling | Enzyme Regulator | Secreted | Widely expressed | CST1 | 16 | 6.8 | - | + |  |  |
| **100** | 1 | Cathepsin B | Immune Response | Enzyme | Cytoplasm | Ubiquitous | CTSB | 38 | 5.9 | + | + |  |  |
| **101** | 1 | Cathepsin D | Immune Response | Enzyme | Cytoplasm | Ubiquitous | CTSD | 45 | 6.1 | + | + |  | 90 |
| **102** | 2 | Cathepsin Z | Immune Response | Enzyme | Cytoplasm | Ubiquitous | CTSZ | 34 | 6.7 | + | + | 54 |  |
| **103** | 1 | Dipeptidyltransferase IV | Cell Adhesion | Enzyme | Cytoplasm | Widely expressed | DPP4 | 88 | 5.7 | + | + |  |  |
| **104** | 2 | Enoyl-CoA hydratase, mitochondrial | Cell Homeostasis | Enzyme | Mitochondrial | Widely expressed | ECHS1 | 31 | 8.3 | - |  |  | 58,83,92 |
| **105** | 3 | Extracellular matrix protein 1 | unspecified | Protein Binding | Secreted | Widely expressed | ECM1 | 61 | 6.3 | + | + |  |  |
| **106** | 3 | Elongation factor 1-alpha 1 | Cell Homeostasis | Binding | Cytoplasm | Widely expressed | EEF1A1 | 49 | 3.8 | - | + |  |  |
| **107** | 3 | Elongation factor 2 | Cell Homeostasis | Enzyme | Cytoplasm | Ubiquitous | EEF2 | 42 | 3.8 | - | + |  |  |
| **108** | 3 | Translation elongation factor 2 | Cell Homeostasis | Binding | Cytoplasm | Ubiquitous | EEF-2 | 95 | 6.4 | - | + |  |  |
| **109** | 1 | Alpha-enolase | Immune Response | Enzyme | Cytoplasm | Blood cells | ENO1 | 47 | 7.0 | + | + | 41,42 | 51 |
| **110** | 1 | Gamma-enolase | Metabolism | Enzyme | Cytoplasm | Widely expressed | ENO2 | 47 | 4.9 | - | + |  |  |
| **111** | 3 | Glutamyl aminopeptidase | Cell Adhesion | Enzyme | Plasma membrane | Widely expressed | ENPEP | 109 | 5.3 | - | + |  |  |
| **112** | 3 | Ectonucleotide pyrophosphatase/phosphodiesterase family member 7 | Metabolism | Enzyme | Plasma membrane | Liver | ENPP7 | 51 | 6.4 | - | + |  |  |
| **113** | 2 | Erlin-2 | Cell Homeostasis | others | ER | Ubiquitous | ERLIN2 | 38 | 5.5 | - | + |  |  |
| **114** | 1 | Endoplasmic reticulum resident protein 29 | Cell Homeostasis | Chaperone | ER | Ubiquitous | ERP29 | 29 | 6.8 | - |  |  | 84 |
| **115** | 2 | Endoplasmic reticulum resident protein 44 | Cell Homeostasis | Chaperone | ER | Ubiquitous | ERP44 | 40 | 6.1 | - | + | + |  |
| **116** | 3 | Junctional adhesion molecule A | Cell Adhesion | Unknown | Cytoplasm | Epithelium | F11R | 32 | 9.8 | - | + |  |  |
| **117** | 3 | Fatty acid-binding protein, liver | Metabolism | Binding | Cytoplasm | Liver | FABP1 | 45 | 5.5 | + | + |  |  |
| **118** | 3 | Protein FAM151A | Unknown | Unknown | Plasma membrane | Unknown | FAM151A | 64 | 6.2 | - | + |  |  |
| **119** | 2 | Fructose-1,6-bisphosphatase 1 | Metabolism | Enzyme | Cytoplasm | Liver | FBP1 | 37 | 6.5 | + | + |  |  |
| **120** | 1 | IgGFc-binding protein | Immune Response | Protein Binding | Secreted | Other GI | FCGBP | 54 | 5.5 | + | + |  |  |
| **121** | 2 | Fibrinogen alpha chain | Blood coagulation | Protein Binding | Secreted | Plasma | FGA | 29 | 5.5 | + | + |  |  |
| **122** | 1 | Fibrinogen beta chain | Blood coagulation | Protein Binding | Secreted | Plasma | FGB | 56 | 8.5 | - | + | 12,38,39 |  |
| **123** | 1 | Fibrinogen Gamma chain isoform, gamma-A precursor | Blood coagulation | Protein Binding | Secreted | Liver | FGG | 52 | 5.4 | + | + | 22,23 |  |
| **124** | 2 | Fibronectin | Blood coagulation | Protein Binding | Secreted | Liver | FN1 | 28 | 5.5 | + | + |  |  |
| **125** | 2 | Fructosamine-3-kinase | Metabolism | Enzyme | Cytoplasm | Blood cells | FN3K | 35 | 7.1 | - | + |  |  |
| **126** | 1 | Glyceraldehyde-3-phosphate dehydrogenase | Metabolism | Enzyme | Cytoplasm | Widely expressed | GAPDH | 36 | 8.6 | - |  | 48,49 |  |
| **127** | 1 | Vitamin D-binding protein | Transport | Binding | Secreted | Widely expressed | GC | 48 | 8.2 | + | + | 55 |  |
| **128** | 1 | Rab GDP dissociation inhibitor beta | Signalling | Enzyme Regulator | Cytoplasm | Ubiquitous | GDI2 | 30 | 3.8 | + | + |  |  |
| **129** | 3 | Glucosamine-fructose-6-phosphate aminotransferase | Metabolism | Enzyme | Cytoplasm | Widely expressed | GFPT1 | 79 | 6.7 | - | + |  |  |
| **130** | 1 | Gamma-glutamyltransferase 1 | Cell Homeostasis | Enzyme | Plasma membrane | Liver | GGT1 | 61 | 6.7 | - | + |  |  |
| **131** | 3 | Growth hormone receptor | Signalling | Protein Binding | Plasma membrane | Liver | GHR | 37 | 9.8 | - | + |  |  |
| **132** | 2 | Pancreatic secretory granule membrane major glycoprotein GP2 | Transport | Binding | Plasma membrane | Pancreas | GP2 | 59 | 5.1 | - | + |  |  |
| **133** | 3 | Glypican-1 | Unknown | Binding | Plasma membrane | Widely expressed | GPC1 | 28 | 5.5 | - | + |  |  |
| **134** | 2 | G-protein coupled receptor 126 | Signalling | Receptor Activity | Plasma membrane | Liver | GPR126 | 137 | 8.2 | + | + |  |  |
| **135** | 2 | G-protein coupled receptor family C group 5 member C | Signalling | Protein Binding | Plasma membrane | Other GI | GPRC5C | 53 | 8.1 | - | + |  |  |
| **136** | 3 | Glutathione peroxidase | Cell Homeostasis | Enzyme | Cytoplasm | Widely expressed | GPX | 22 | 6.2 | + | + |  |  |
| **137** | 2 | Gelsolin | Cytoskeleton | Enzyme/ Protein binding | Cytoplasm | Blood cells | GSN | 86 | 5.9 | + | + |  |  |
| **138** | 2 | Glutathione synthetase | Cell Homeostasis | Enzyme | Cytoplasm | Ubiquitous | GSS | 52 | 5.7 | + | + |  |  |
| **139** | 1 | Glutathione S-transferase P | Metabolism | Enzyme | Cytoplasm | Widely expressed | GSTP1 | 23 | 5.4 | - |  | 71,73 | 20,60?,89? |
| **140** | 3 | Haemoglobin subunit alpha | Transport | Binding | Cytoplasm | Blood cells | HBA1 | 15 | 8.7 | - | + | 1, 102 |  |
| **141** | 1 | Haemoglobin, subunit beta | Transport | Binding | Cytoplasm | Blood cells | HBB | 16 | 6.7 | + | + | 40,64,84,97,98,99, 100 |  |
| **142** | 1 | Haemoglobin, subunit delta | Transport | Binding | Cytoplasm | Blood cells | HBD | 16 | 7.8 | - | + |  |  |
| **143** | 1 | Haemoglobin subunit gamma-2 | Transport | Binding | Cytoplasm | Blood cells | HBG2 | 16 | 6.6 | - | + |  |  |
| **144** | 3 | Histone H2A type 1 | Cell Homeostasis | Binding | Nucleus | Ubiquitous | HIST1H2AI | 14 | 10.9 | - | + |  |  |
| **145** | 3 | Histone H3.1 | Cell Homeostasis | Binding | Nucleus | Ubiquitous | HIST1H3A | 66 | 6.9 | - | + |  |  |
| **146** | 3 | Histone H2A type 2-A | Cell Homeostasis | Binding | Nucleus | Ubiquitous | HIST2H2AA3 | 74 | 6.9 | - | + |  |  |
| **147** | 2 | Heterogeneous nuclear ribonucleoprotein A2/B1 | Cell Homeostasis | others | Cytoplasm | Ubiquitous | HNRNPA2B1 | 37 | 9.0 | - |  |  |  |
| **148** | 1 | Haptoglobin | Transport | Protein Binding | Secreted | Liver | HP | 45 | 6.1 | + | + | 27,29,32,33,85,86,87,88,89 | 15,19 |
| **149** | 2 | Hypoxanthine-guanine phosphoribosyltransferase | Cell Homeostasis | Enzyme | Cytoplasm | Widely expressed | HPRT1 | 25 | 6.2 | - | + |  |  |
| **150** | 1 | Hemopexin | Transport | Protein Binding | Cytoplasm | Liver | HPX | 52 | 6.6 | + | + |  |  |
| **151** | 2 | Histidine-rich glycoprotein | unspecified | Binding | Secreted | Liver | HRG | 60 | 7.1 | + | + |  |  |
| **152** | 3 | Hornerin | unspecified | Protein Binding | Cytoplasm | Others | HRNR | 282 | 10.0 | - | + |  |  |
| **153** | 1 | Heat shock protein HSP 90-alpha | Immune Response | Chaperone | Cytoplasm | Widely expressed | HSP90AA1 | 85 | 4.9 | - | + |  |  |
| **154** | 1 | Heat shock protein HSP 90-beta | Immune Response | Chaperone | Cytoplasm | Widely expressed | HSP90AB1 | 83 | 5.0 | + | + |  |  |
| **155** | 1 | Heat shock 70 kDa protein 1A/1B | Immune Response | Chaperone | Cytoplasm | Widely expressed | HSPA1A | 70 | 5.5 | - |  | 20 |  |
| **156** | 3 | 78 kDa glucose-regulated protein | unspecified | Chaperone | ER | Ubiquitous | HSPA5 | 72 | 5.1 | + | + |  |  |
| **157** | 1 | Heat shock cognate 71 kDa protein | Immune Response | Chaperone | Cytoplasm | Widely expressed | HSPA8 | 71 | 5.4 | + | + |  |  |
| **158** | 3 | Heat shock protein beta-1 | Immune Response | Chaperone | Cytoplasm | Widely expressed | HSPB1 | 23 | 6.0 | - | + |  |  |
| **159** | 3 | 10 kDa heat shock protein, mitochondrial | Immune Response | Chaperone | Mitochondrial | Ubiquitous | HSPE1 | 11 | 8.9 | - | + |  |  |
| **160** | 1 | Intercellular adhesion molecule 1 | Cell Adhesion | Protein Binding | Plasma membrane | Widely expressed | ICAM1 | 58 | 8.3 | + | + |  |  |
| **161** | 1 | Isocitrate dehydrogenase [NADP] cytoplasmic | Cell Homeostasis | Enzyme | Cytoplasm | Widely expressed | IDH1 | 47 | 6.5 | + |  | 53 |  |
| **162** | 1 | Interferon alpha-inducible protein 6 | Immune Response | Protein Binding | Plasma membrane | Widely expressed | IFI6 | 39 | 5.5 | - | + |  |  |
| **163** | 1 | Ig alpha-1 chain C region | Immune Response | Protein Binding | Secreted | Blood cells | IGHA1 | 40 | 6.7 | + | + |  |  |
| **164** | 3 | Ig alpha-2 chain C region | Immune Response | Protein Binding | Secreted | Blood cells | IGHA2 | 37 | 5.7 | + | + |  |  |
| **165** | 3 | Ig epsilon chain C region | Immune Response | Protein Binding | Secreted | Blood cells | IGHE | 47 | 8.4 | - | + |  |  |
| **166** | 3 | Ig gamma-1 chain C region | Immune Response | Protein Binding | Secreted | Blood cells | IGHG1 | 36 | 8.5 | - | + |  |  |
| **167** | 1 | Ig gamma-2 chain C region | Immune Response | Protein Binding | Secreted | Blood cells | IGHG2 | 36 | 7.7 | - | + |  |  |
| **168** | 1 | Ig gamma-3 chain C region | Immune Response | Protein Binding | Secreted | Blood cells | IGHG3 | 41 | 8.2 | - |  | 36 |  |
| **169** | 1 | Ig mu chain C region | Immune Response | Protein Binding | Secreted | Blood cells | IGHM | 49 | 6.4 | - | + |  |  |
| **170** | 1 | Ig kappa chain C region | Immune Response | Protein Binding | Secreted | Blood cells | IGKC | 12 | 5.6 | - | + |  |  |
| **171** | 1 | Ig lambda-1 chain C regions | Immune Response | Protein Binding | Secreted | Blood cells | IGLC1 | 11 | 7.9 | + |  | 59 |  |
| **172** | 3 | Interleukin-6 receptor subunit beta | Immune Response | Receptor Activity | Plasma membrane | Ubiquitous | IL6ST | 104 | 5.6 | + | + |  |  |
| **173** | 3 | Integrin alpha-1 | Cell Adhesion | Protein Binding | Plasma membrane | Widely expressed | ITGA1 | 131 | 5.9 | - | + |  |  |
| **174** | 3 | Integrin beta 1 | Cell Adhesion | Protein Binding | Plasma membrane | Widely expressed | ITGB1 | 88 | 5.3 | + | + |  |  |
| **175** | 1 | Inter-alpha-trypsin inhibitor heavy chain H1 | Protease Inhibitor | Enzyme Regulator | Secreted | Liver | ITIH1 | 101 | 6.3 | + | + |  |  |
| **176** | 3 | Inter-alpha-trypsin inhibitor heavy chain H2 | Protease Inhibitor | Enzyme Regulator | Secreted | Liver | ITIH2 | 106 | 6.4 | + | + |  |  |
| **177** | 1 | Inter-alpha-trypsin inhibitor heavy chain H4 | Protease Inhibitor | Enzyme Regulator | Secreted | Liver | ITIH4 | 48 | 6.7 | + | + |  |  |
| **178** | 2 | Kininogen-1 | Immune Response | Enzyme Regulator | Secreted | Widely expressed | KNG1 | 72 | 6.3 | + | + |  |  |
| **179** | 3 | Keratin, type II cytoskeletal 1 | Cytoskeleton | Protein Binding | Cytoplasm | Others | KRT1 | 66 | 8.2 | - |  |  | 22,28,74,87 |
| **180** | 3 | Keratin, type I cytoskeletal 10 | Cytoskeleton | Protein Binding | Cytoplasm | Others | KRT10 | 59 | 5.1 | - |  |  |  |
| **181** | 3 | Keratin, type II cytoskeletal 2 epidermal | Cytoskeleton | Protein Binding | Cytoplasm | Others | KRT2 | 65 | 8.1 | + |  |  | 78 |
| **182** | 3 | Keratin, type II cytoskeletal 8 | Cytoskeleton | Protein Binding | Cytoplasm | Unknown | KRT8 | 54 | 5.5 | - |  |  | 1 |
| **183** | 3 | Keratin, type II cytoskeletal 9 | Cytoskeleton | Protein Binding | Cytoplasm | Others | KRT9 | 62 | 5.1 | + |  |  | 4 |
| **184** | 3 | LIM and SH3 domain protein 1 | Cytoskeleton | Protein Binding | Cytoplasm | Widely expressed | LASP1 | 30 | 6.6 | - | + |  |  |
| **185** | 2 | Neutrophil gelatinase-associated lipocalin | Transport | Protein Binding | Secreted | Widely expressed | LCN2 | 35 | 5.5 | + | + |  |  |
| **186** | 2 | Plastin-2 | Cytoskeleton | Binding | Cytoplasm | Others | LCP1 | 70 | 5.2 | - | + |  |  |
| **187** | 1 | L-lactate dehydrogenase A chain | Cell Homeostasis | Enzyme | Cytoplasm | Widely expressed | LDHA | 37 | 8.4 | + | + |  |  |
| **188** | 3 | L-lactate dehydrogenase B chain | Cell Homeostasis | Enzyme | Cytoplasm | Ubiquitous | LDHB | 37 | 5.7 | + | + |  |  |
| **189** | 3 | Galectin-3 | Cell Adhesion | Protein Binding | Nucleus | Other GI | LGALS3 | 46 | 5.5 | - | + |  |  |
| **190** | 1 | Galectin-3-binding protein | Cell Adhesion | Protein Binding | Secreted | Ubiquitous | LGALS3BP | 65 | 5.1 | + | + |  |  |
| **191** | 3 | Galectin-4 | Metabolism | Binding | Cytoplasm | Other GI | LGALS4 | 46 | 5.5 | - | + |  |  |
| **192** | 2 | Gastric triacylglycerol lipase | Metabolism | Enzyme | Secreted | Other GI | LIPF | 45 | 6.8 | - | + |  |  |
| **193** | 1 | Lamin-A/C | Cytoskeleton | Protein Binding | Nucleus | Widely expressed | LMNA | 74 | 6.6 | - | + |  |  |
| **194** | 2 | Lamin-B2 | Cytoskeleton | Protein Binding | Nucleus | Widely expressed | LMNB2 | 68 | 5.3 | - | + |  |  |
| **195** | 3 | Leucyl-cystinyl aminopeptidase | Signalling | Enzyme | Plasma membrane | Widely expressed | LNPEP | 117 | 5.5 | - | + |  |  |
| **196** | 3 | Leucine-rich alpha-2-glycoprotein | Signalling | Protein Binding | Secreted | Others | LRG1 | 38 | 6.5 | + | + |  |  |
| **197** | 1 | Lactotransferrin | Transport | Binding | Secreted | Widely expressed | LTF | 38 | 5.5 | - | + |  |  |
| **198** | 3 | Lumican | Cytoskeleton | Protein Binding | Secreted | Widely expressed | LUM | 38 | 6.2 | + |  | + |  |
| **199** | 3 | Lysozyme C | Immune Response | Enzyme | Secreted | Epithelium | LYZ | 17 | 9.4 | - | + |  |  |
| **200** | 1 | Malate dehydrogenase, cytoplasmic | Cell Homeostasis | Enzyme | Cytoplasm | Widely expressed | MDH1 | 36 | 6.9 | + | + | 60 |  |
| **201** | 1 | Neprilysin | Metabolism | Enzyme | Plasma membrane | Others | MME | 86 | 5.7 | - | + |  |  |
| **202** | 2 | Matrilysin | Unknown | Enzyme | Secreted | Widely expressed | MMP7 | 30 | 7.7 | - | + |  |  |
| **203** | 3 | Neutrophil collagenase | Immune Response | Enzyme | Secreted | Blood cells | MMP8 | 53 | 6.4 | + | + |  |  |
| **204** | 2 | Matrix metalloproteinase-9 | Immune Response | Enzyme | Secreted | Blood cells | MMP9 | 78 | 5.7 | + | + |  |  |
| **205** | 3 | 39S ribosomal protein L3, mitochondrial | Cell Homeostasis | Binding | Mitochondrial | Widely expressed | MRPL3 | 51 | 9.5 | - | + |  |  |
| **206** | 1 | Mucin-5B | Immune Response | Protein Binding | Secreted | Others | MUC5B | 590 | 6.2 | - |  | + |  |
| **207** | 3 | Mucin-6 | Immune Response | Protein Binding | Secreted | Other GI | MUC6 | 252 | 7.0 | - |  | + |  |
| **208** | 3 | Myosin-9 | Cytoskeleton | Protein Binding | Cytoplasm | Widely expressed | MYH9 | 45 | 6.5 | + | + |  |  |
| **209** | 2 | NADH dehydrogenase [ubiquinone] iron-sulphur protein 3, mitochondrial | Cell Homeostasis | Enzyme | Mitochondrial | Widely expressed | NDUFS3 | 30 | 7.0 | - |  |  | 77 |
| **210** | 3 | Nucleoside diphosphate kinase A | Metabolism | Enzyme | Cytoplasm | Ubiquitous | NME1 | 17 | 5.8 | - | + |  |  |
| **211** | 3 | Nucleoside diphosphate kinase B | Metabolism | Enzyme | Cytoplasm | Ubiquitous | NME2 | 17 | 8.5 | - | + |  |  |
| **212** | 1 | Neuropilin-1 | Unknown | Receptor Activity | Secreted | Widely expressed | NRP1 | 103 | 5.6 | - | + |  |  |
| **213** | 1 | 5'-nucleotidase | Cell Homeostasis | Enzyme | Plasma membrane | Widely expressed | NT5E | 63 | 6.6 | - | + |  |  |
| **214** | 3 | 2-oxoglutarate dehydrogenase, mitochondrial | Cell Homeostasis | Enzyme | Mitochondrial | Widely expressed | OGDH | 116 | 6.4 | - | + |  |  |
| **215** | 1 | Alpha-1-acid glycoprotein 1 | Immune Response | Unknown | Secreted | Liver | ORM1 | 24 | 4.9 | + | + |  |  |
| **216** | 1 | Alpha-1-acid glycoprotein 2 | Immune Response | Unknown | Secreted | Liver | ORM2 | 24 | 5.0 | + |  | 34 |  |
| **217** | 3 | RNA-directed RNA polymerase catalytic subunit | Cell Homeostasis | Enzyme | Cytoplasm | Unknown | P2 | 12 | 6.0 | - |  |  | 11 |
| **218** | 1 | PDI | Cell Homeostasis | Enzyme | ER | Widely expressed | P4HB | 37 | 7.2 | + |  |  |  |
| **219** | 2 | Protein DJ-1 | Immune Response | Chaperone | Cytoplasm | Ubiquitous | PARK7 | 20 | 6.3 | + |  |  | 67,69,71 |
| **220** | 1 | Programmed cell death protein 10 | Immune Response | Protein Binding | Cytoplasm | Ubiquitous | PDCD10 | 45 | 3.9 | - | + |  |  |
| **221** | 3 | Protein disulfide-isomerase A3 | Cell Homeostasis | Enzyme | ER | Widely expressed | PDIA3 | 57 | 6.0 | + |  | 90 |  |
| **222** | 1 | Protein disulfide isomerase-associated 4 | Cell Homeostasis | Enzyme | ER | Unknown | PDIA4 | 41 | 3.8 | + | + |  |  |
| **223** | 1 | Phosphatidylethanolamine-binding protein 1 | Unknown | Enzyme Regulator | Cytoplasm | Unknown | PEBP1 | 21 | 7.0 | - | + | 83 |  |
| **224** | 1 | Phosphoglycerate mutase 1 | Cell Homeostasis | Enzyme | Cytoplasm | Widely expressed | PGAM1 | 37 | 6.0 | - | + |  |  |
| **225** | 1 | 6-phosphogluconate dehydrogenase, decarboxylating | Cell Homeostasis | Enzyme | Cytoplasm | Widely expressed | PGD | 29 | 4.3 | + | + |  |  |
| **226** | 1 | Phosphoglycerate kinase 1 | Cell Homeostasis | Enzyme | Cytoplasm | Widely expressed | PGK1 | 30 | 9.2 | + |  | 46 |  |
| **227** | 1 | 6-phosphogluconolactonase | Metabolism | Enzyme | Cytoplasm | Liver | PGLS | 28 | 5.7 | - | + |  |  |
| **228** | 1 | Prohibitin | Cell Homeostasis | Enzyme Regulator | Mitochondrial | Widely expressed | PHB | 36 | 7.2 | - | + |  |  |
| **229** | 3 | Peptidase inhibitor 15 | Metabolism | Enzyme Regulator | Secreted | Widely expressed | PI15 | 29 | 8.4 | - | + |  |  |
| **230** | 1 | Polymeric immunoglobulin receptor | Immune Response | Protein Binding | Plasma membrane | Widely expressed | PIGR | 83 | 5.6 | + | + |  |  |
| **231** | 2 | Pyruvate kinase isozymes R/L | Metabolism | Binding | Cytoplasm | Liver | PKLR | 51 | 11.0 | + | + |  |  |
| **232** | 3 | Phospholipase A2 | Metabolism | Enzyme | Secreted | Pancreas | PLA2G1B | 16 | 8.2 | - | + |  |  |
| **233** | 3 | Platelet-activating factor acetylhydrolase | Metabolism | Enzyme | Secreted | Plasma | PLA2G7 | 36 | 6.0 | + | + |  |  |
| **234** | 2 | Plasminogen | Blood coagulation | Enzyme | Secreted | Liver | PLG | 91 | 7.1 | + | + |  |  |
| **235** | 1 | Plastin-1 | Cytoskeleton | Protein Binding | Cytoplasm | Other GI | PLS1 | 31 | 6.0 | - | + |  |  |
| **236** | 1 | Plastin-3 | Cytoskeleton | Protein Binding | Cytoplasm | Widely expressed | PLS3 | 31 | 6.0 | + | + |  |  |
| **237** | 2 | Pancreatic triacylglycerol lipase | Metabolism | Enzyme | Secreted | Pancreas | PNLIP | 51 | 6.3 | - |  | + |  |
| **238** | 3 | Serum paraoxonase/arylesterase 1 | Metabolism | Enzyme | Secreted | Liver | PON1 | 40 | 5.1 | + | + |  |  |
| **239** | 1 | POTE ankyrin domain family member E | Unknown | Protein Binding | Plasma membrane | Widely expressed | POTEE | 121 | 5.8 | - | + |  |  |
| **240** | 3 | Pyrophosphatase 1 | Metabolism | Enzyme | Cytoplasm | Widely expressed | PPA1 | 28 | 6.0 | - | + |  |  |
| **241** | 1 | Peroxiredoxin-1 | Cell Homeostasis | Enzyme | Cytoplasm | Widely expressed | PRDX1 | 22 | 8.3 | + |  | 81 | 81 |
| **242** | 3 | Peroxiredoxin-2 | Cell Homeostasis | Enzyme | Cytoplasm | Unknown | PRDX2 | 22 | 5.7 | - |  | 74,77 | 21 |
| **243** | 3 | Thioredoxin-dependent peroxide reductase, mitochondrial  (Peroxiredoxin-3) | Cell Homeostasis | Enzyme | Mitochondrial | Widely expressed | PRDX3 | 28 | 7.7 | - |  |  | 68 |
| **244** | 3 | Peroxiredoxin-4 | Cell Homeostasis | Enzyme | Cytoplasm | Widely expressed | PRDX4 | 31 | 5.9 | - |  |  | 80 |
| **245** | 1 | Peroxiredoxin-6 | Cell Homeostasis | Enzyme | Cytoplasm | Widely expressed | PRDX6 | 25 | 6.0 | - |  | 70 |  |
| **246** | 2 | Trypsin-1 | Metabolism | Enzyme | Secreted | Pancreas | PRSS1 | 27 | 6.1 | + | + |  |  |
| **247** | 2 | Trypsin-3 | Metabolism | Enzyme | Secreted | Pancreas | PRSS3 | 44 | 10.2 | + | + |  |  |
| **248** | 1 | Proteasome activator complex subunit 1 | Immune Response | Unknown | Cytoplasm | Unknown | PSME1 | 29 | 5.8 | - |  |  | 85,88,91 |
| **249** | 3 | Proteasome activator complex subunit 2 | Cell Homeostasis | Enzyme Regulator | Cytoplasm | Unknown | PSME2 | 46 | 4.6 | - | + |  |  |
| **250** | 1 | Prostaglandin reductase 1 | Immune Response | Enzyme | Cytoplasm | Liver | PTGR1 | 43 | 10.2 | - | + |  |  |
| **251** | 3 | Prostaglandin reductase 2 | Immune Response | Enzyme | Cytoplasm | Widely expressed | PTGR2 | 38 | 5.3 | - |  | 28,29 |  |
| **252** | 3 | Tyrosine-protein phosphatase non-receptor type 1 | Signalling | Enzyme | ER | Unknown | PTPN1 | 50 | 5.9 | - | + |  |  |
| **253** | 3 | Glycogen phosphorylase, brain form | Metabolism | Enzyme | Cytoplasm | Liver | PYGB | 97 | 6.40 | - | + |  |  |
| **254** | 2 | Ras-related protein Rab-11B | Cell Homeostasis | Binding | ER | Ubiquitous | RAB11B | 24 | 5.6 | - | + |  |  |
| **255** | 3 | Ras-related protein Rab-15 | Transport | Binding | Plasma membrane | Ubiquitous | RAB15 | 24 | 5.5 | - | + |  |  |
| **256** | 3 | Ras-related protein Rab-1A | Transport | Binding | ER | Ubiquitous | RAB1A | 23 | 5.9 | - | + |  |  |
| **257** | 3 | Ras-related protein Rab-35 | Transport | Protein Binding | Plasma membrane | Ubiquitous | RAB35 | 23 | 8.5 | - | + |  |  |
| **258** | 3 | Ras-related protein Rab-8B | Transport | Protein Binding | Plasma membrane | Ubiquitous | RAB8B | 24 | 9.2 | - | + |  |  |
| **259** | 3 | 60S acidic ribosomal protein P0 | Cell Homeostasis | Binding | Cytoplasm | Widely expressed | RPLP0 | 34 | 5.7 | - | + |  |  |
| **260** | 3 | Ubiquitin | Cell Homeostasis | Protein Binding | Cytoplasm | Ubiquitous | RPS27A | 9 | 6.56 | - | + |  |  |
| **261** | 3 | 40S ribosomal protein SA | Cell Homeostasis | Binding | Cytoplasm | Ubiquitous | RPSA | 33 | 4.79 | - | + |  |  |
| **262** | 3 | Reticulon 4 receptor-like 2 | Unknown | Receptor Activity | Plasma membrane | Liver | RTN4RL2 | 66 | 12.0 | + | + |  |  |
| **263** | 3 | Protein S100-A4 | Signalling | Binding | Nucleus | Ubiquitous | S100A4 | 12 | 5.9 | - | + |  |  |
| **264** | 3 | Protein S100-A6 | Signalling | Binding | Nucleus | Widely expressed | S100A6 | 10 | 5.3 | - | + |  |  |
| **265** | 1 | Protein S100-A8 | Immune Response | Binding | Plasma membrane | Epithelium | S100A8 | 11 | 6.5 | + | + | 101 |  |
| **266** | 2 | Protein S100-A9 | Immune Response | Binding | Plasma membrane | Epithelium | S100A9 | 13 | 5.7 | + | + | 103 |  |
| **267** | 3 | Serum amyloid A-4 protein | Immune Response | others | Secreted | Liver | SAA4 | 15 | 9.2 | + | + |  |  |
| **268** | 2 | Selenium-binding protein 1 | Transport | Protein Binding | Cytoplasm | Liver | SELENBP1 | 80 | 3.79 | + | + |  |  |
| **269** | 1 | Alpha-1-antitrypsin | Protease Inhibitor | Enzyme Regulator | Secreted | Liver | SERPINA1 | 47 | 5.4 | + | + | 35 | 46 |
| **270** | 1 | Alpha-1-antichymotrypsin | Immune Response | Enzyme Regulator | Secreted | Liver | SERPINA3 | 79 | 5.72 | + | + |  |  |
| **271** | 3 | Kallistatin | Signalling | Enzyme Regulator | Secreted | Liver | SERPINA4 | 49 | 7.3 | + | + |  |  |
| **272** | 1 | Plasma serine protease inhibitor | Blood coagulation | Enzyme Regulator | Secreted | Liver | SERPINA5 | 46 | 9.3 | + | + |  |  |
| **273** | 2 | Corticosteroid-binding globulin | Signalling | Binding | Secreted | Liver | SERPINA6 | 45 | 5.64 | + | + |  |  |
| **274** | 3 | Leukocyte elastase inhibitor | Immune Response | Enzyme Regulator | Cytoplasm | Widely expressed | SERPINB1 | 43 | 5.9 | + | + |  |  |
| **275** | 1 | Serpin B6 | Blood coagulation | Enzyme Regulator | Cytoplasm | Widely expressed | SERPINB6 | 43 | 5.2 | - | + |  |  |
| **276** | 2 | Alpha-2-antiplasmin | Blood coagulation | Enzyme Regulator | Secreted | Liver | SERPINF2 | 86 | 5.72 | + | + |  |  |
| **277** | 1 | plasma protease (C1) inhibitor | Immune Response | Enzyme Regulator | Secreted | Liver | SERPING1 | 55 | 6.1 | + | + |  |  |
| **278** | 1 | 14-3-3 protein sigma | Signalling | Protein Binding | Cytoplasm | Epithelium | SFN | 28 | 4.7 | - | + |  |  |
| **279** | 3 | Tyrosine-protein phosphatase non-receptor type substrate 1 | Cell Adhesion | Binding | Plasma membrane | Ubiquitous | SIRPA | 54 | 9.47 | - | + |  |  |
| **280** | 3 | Choline transporter-like protein 4 | Transport | Protein Binding | Plasma membrane | Widely expressed | SLC44A4 | 79 | 8.91 | - | + |  |  |
| **281** | 2 | Cu/Zn superoxide dismutase | Cell Homeostasis | Enzyme | Cytoplasm | Widely expressed | SOD1 | 29 | 5.57 | + | + |  |  |
| **282** | 3 | Superoxide dismutase [Mn], mitochondrial | Cell Homeostasis | Enzyme | Mitochondrial | Widely expressed | SOD2 | 25 | 8.4 | - | + |  |  |
| **283** | 2 | Sorbitol dehydrogenase | Metabolism | Enzyme | Cytoplasm | Widely expressed | SORD | 38 | 8.23 | - | + |  |  |
| **284** | 3 | Erythrocyte band 7 integral membrane protein | Signalling | Protein Binding | Plasma membrane | Widely expressed | STOM | 32 | 7.71 | - | + |  |  |
| **285** | 2 | GTP-specific succinyl-CoA synthetase beta subunit | Cell Homeostasis | Enzyme | Mitochondrial | Widely expressed | SUCLG2 | 47 | 6.2 | - | + |  |  |
| **286** | 3 | Transgelin | Unknown | Protein Binding | Cytoplasm | Others | TAGLN | 23 | 8.87 | - | + |  |  |
| **287** | 2 | Transcobalamin-1 | Transport | Binding | Secreted | Blood cells | TCN1 | 33 | 5.18 | - |  | + |  |
| **288** | 1 | Transferrin | Transport | Binding | Secreted | Liver | TF | 79 | 6.8 | + | + | 3,4,5,8,9,10,11,63 |  |
| **289** | 3 | Trefoil factor 2 | Metabolism | Receptor Activity | Secreted | Pancreas | TFF2 | 41 | 5.19 | - | + |  |  |
| **290** | 3 | Transketolase | Metabolism | Enzyme | Cytoplasm | Unknown | TKT | 35 | 8.41 | + | + |  |  |
| **291** | 3 | Transmembrane protease serine 6 | Unknown | Enzyme | Plasma membrane | Liver | TMPRSS6 | 90 | 6.3 | - | + |  |  |
| **292** | 1 | Triosephosphate isomerase | Metabolism | Enzyme | Cytoplasm | Ubiquitous | TPI1 | 27 | 6.5 | + |  | 66 |  |
| **293** | 1 | Tropomyosin alpha-1 chain | Cytoskeleton | Protein Binding | Cytoplasm | Widely expressed | TPM1 | 33 | 4.7 | + |  |  | 30 |
| **294** | 3 | Tropomyosin alpha-3 chain | Cytoskeleton | Protein Binding | Cytoplasm | Widely expressed | TPM3 | 33 | 4.7 | - | + |  |  |
| **295** | 2 | Heat shock protein 75 kDa, mitochondrial | Immune Response | Chaperone | Cytoplasm | Widely expressed | TRAP1 | 80 | 8.3 | - | + |  |  |
| **296** | 1 | Transthyretin | Transport | Protein Binding | Secreted | Liver | TTR | 16 | 5.52 | + | + |  |  |
| **297** | 3 | Thioredoxin domain-containing protein 12 | Cell Homeostasis | Enzyme | ER | Widely expressed | TXNDC12 | 56 | 3.8 | - | + |  |  |
| **298** | 2 | Thioredoxin reductase 1, cytoplasmic | Cell Homeostasis | Enzyme | Cytoplasm | Widely expressed | TXNRD1 | 71 | 7.2 | - | + |  |  |
| **299** | 1 | Pantetheinase | Cell Homeostasis | Enzyme | Plasma membrane | Liver | VNN1 | 35 | 5.49 | + | + |  |  |
| **300** | 2 | Aminopeptidase-P | Signalling | Enzyme | Cytoplasm | Widely expressed | XPNPEP1 | 58 | 6.4 | - | + |  |  |
| **301** | 1 | 14-3-3 protein beta/alpha | Signalling | Enzyme | Cytoplasm | Ubiquitous | YWHAB | 69 | 6.74 | + | + |  |  |
|  |  |  |  |  |  |  |  |  |  |  |  |  |  |
|  | ^1^ 1>400; 2=200-400; 3 100-200 | |  |  |  |  |  |  |  |  |  |  |  |
|  | ^2^ Qian et al., Sheng et al. | |  |  |  |  |  |  |  |  |  |  |  |
