# Supplementary material for: S100A9 is a Biliary Protein Marker of Disease Activity in Primary Sclerosing Cholangitis
Source: PLoS One. 2012 Jan 11;7(1):e29821. doi: 10.1371/journal.pone.0029821 (PMC3256182; doi:10.1371/journal.pone.0029821)
Supplement: Table S2 — List of predicted bile duct-derived bile proteins separated by a Mascot score >100 or <100. (DOCX) [file pone.0029821.s002.docx]

**Supplemental Table 2**

List of predicted bile duct-derived bile proteins separated by a Mascot score > 100 or < 100.

| **Mascot scores >100** | | |  |
| --- | --- | --- | --- |
|  |  |  |  |
| **No.** | **Score; 1>400; 2=200-400; 3 100-200** | **Protein name** | **Accession #(GI:)** |
| **1** | 2 | hCG1640852 | 119604226 |
| **2** | 1 | hCG1811473 | 119619074 |
| **3** | 2 | hCG20174, isoform CRA_b | 119626933 |
| **4** | 3 | hCG22067 | 119572363 |
| **5** | 1 | Hypothetical protein gi\|34364641 | 34364641 |
| **6** | 1 | Hypothetical protein gi\|34364643 | 34364643 |
| **7** | 2 | Hypothetical protein gi\|34365085 | 34365085 |
| **8** | 1 | Hypothetical protein gi\|34365139 | 34365139 |
| **9** | 2 | Hypothetical protein gi\|34365166 | 34365166 |
| **10** | 1 | Hypothetical protein gi\|34365168 | 34365168 |
| **11** | 1 | Hypothetical protein gi\|5817160 | 5817160 |
| **12** | 3 | Hypothetical protein gi\|60219520 | 60219520 |
| **13** | 1 | Hypothetical protein gi\|6807647 | 6807647 |
| **14** | 1 | Hypothetical protein isoform 4 | 113408761 |
| **15** | 2 | Hypothetical protein LOC51237 | 117938314 |
| **16** | 1 | Putative gi\|553734 | 553734 |
| **17** | 2 | Unknown (protein for MGC:32654) | 21410211 |
| **18** | 3 | Unknown (protein for MGC:71261) | 40353009 |
| **19** | 1 | Unknown (protein for MGC:88814) | 49257472 |
| **20** | 3 | Unnamed protein product gi\|1335344 | 1335344 |
| **21** | 1 | Unnamed protein product gi\|14042015 | 14042015 |
| **22** | 1 | Unnamed protein product gi\|158259769 | 158259769 |
| **23** | 2 | Unnamed protein product gi\|16550719 | 16550719 |
| **24** | 1 | Unnamed protein product gi\|16554039 | 16554039 |
| **25** | 3 | Unnamed protein product gi\|18676733 | 18676733 |
| **26** | 2 | Unnamed protein product gi\|189066632 | 189066632 |
| **27** | 1 | Unnamed protein product gi\|21757066 | 21757066 |
| **28** | 1 | Unnamed protein product gi\|21758945 | 21758945 |
| **29** | 2 | Unnamed protein product gi\|28317 | 28317 |
| **30** | 3 | Unnamed protein product gi\|28332 | 28332 |
| **31** | 1 | Unnamed protein product gi\|28334 | 28334 |
| **32** | 3 | Unnamed protein product gi\|28404 | 28404 |
| **33** | 1 | Unnamed protein product gi\|28590 | 28590 |
| **34** | 3 | Unnamed protein product gi\|28678 | 28678 |
| **35** | 3 | Unnamed protein product gi\|28738 | 28738 |
| **36** | 3 | Unnamed protein product gi\|28940 | 28940 |
| **37** | 1 | Unnamed protein product gi\|29446 | 29446 |
| **38** | 1 | Unnamed protein product gi\|29501 | 29501 |
| **39** | 1 | Unnamed protein product gi\|31092 | 31092 |
| **40** | 1 | Unnamed protein product gi\|31170 | 31170 |
| **41** | 3 | unnamed protein product gi\|31189 | 31189 |
| **42** | 3 | Unnamed protein product gi\|31283 | 31283 |
| **43** | 2 | Unnamed protein product gi\|32097 | 32097 |
| **44** | 3 | Unnamed protein product gi\|32111 | 32111 |
| **45** | 1 | Unnamed protein product gi\|32486 | 32486 |
| **46** | 1 | Unnamed protein product gi\|33451 | 33451 |
| **47** | 2 | Unnamed protein product gi\|34039 | 34039 |
| **48** | 3 | Unnamed protein product gi\|34228 | 34228 |
| **49** | 1 | Unnamed protein product gi\|34412 | 34412 |
| **50** | 3 | Unnamed protein product gi\|34526063 | 34526063 |
| **51** | 1 | Unnamed protein product gi\|34526163 | 34526163 |
| **52** | 1 | Unnamed protein product gi\|34526220 | 34526220 |
| **53** | 3 | Unnamed protein product gi\|34526424 | 34526424 |
| **54** | 1 | Unnamed protein product gi\|34527233 | 34527233 |
| **55** | 1 | Unnamed protein product gi\|34527275 | 34527275 |
| **56** | 1 | Unnamed protein product gi\|34527290 | 34527290 |
| **57** | 1 | Unnamed protein product gi\|34527413 | 34527413 |
| **58** | 1 | Unnamed protein product gi\|34527453 | 34527453 |
| **59** | 1 | Unnamed protein product gi\|34527679 | 34527679 |
| **60** | 1 | Unnamed protein product gi\|34527698 | 34527698 |
| **61** | 1 | Unnamed protein product gi\|34527739 | 34527739 |
| **62** | 1 | Unnamed protein product gi\|34529647 | 34529647 |
| **63** | 1 | Unnamed protein product gi\|34531724 | 34531724 |
| **64** | 1 | Unnamed protein product gi\|34532520 | 34532520 |
| **65** | 1 | Unnamed protein product gi\|34532522 | 34532522 |
| **66** | 1 | Unnamed protein product gi\|34535785 | 34535785 |
| **67** | 1 | Unnamed protein product gi\|34535866 | 34535866 |
| **68** | 1 | Unnamed protein product gi\|34536020 | 34536020 |
| **69** | 2 | Unnamed protein product gi\|34536114 | 34536114 |
| **70** | 3 | Unnamed protein product gi\|34628 | 34628 |
| **71** | 1 | Unnamed protein product gi\|34711 | 34711 |
| **72** | 2 | Unnamed protein product gi\|34719 | 34719 |
| **73** | 1 | Unnamed protein product gi\|35222 | 35222 |
| **74** | 1 | Unnamed protein product gi\|35897 | 35897 |
| **75** | 2 | Unnamed protein product gi\|36573 | 36573 |
| **76** | 3 | Unnamed protein product gi\|7020017 | 7020017 |
| **77** | 3 | Unnamed protein product gi\|7020171 | 7020171 |
| **78** | 2 | Unnamed protein product gi\|7020203 | 7020203 |
|  |  |  |  |
|  |  |  |  |
| **Mascot Scores<=100** | | |  |
|  | **Mascot Score** | **Protein name** | **Accession #(GI:)** |
| **1** | 32 | hCG1645061 | 119619423 |
| **2** | 47 | hCG1646871 | 119599627 |
| **3** | 47 | hCG1646871 | 119599627 |
| **4** | 46 | hCG1647491 | 119577272 |
| **5** | 46 | hCG1647779 | 119622684 |
| **6** | 31 | hCG1744891 | 119600845 |
| **7** | 33 | hCG1805892 |  |
| **8** | 33 | hCG1808204 |  |
| **9** | 81 | hCG1988300 |  |
| **10** | 35 | hCG2011852 | 119629468 |
| **11** | 33 | hCG2040410 | 119614235 |
| **12** | 32 | hCG2041547 |  |
| **13** | 34 | hCG2041782 |  |
| **14** | 54 | hCG2045741 |  |
| **15** | 33 | Hypothetical protein gi\|169177171 | 169177171 |
| **16** | 44 | Hypothetical protein gi\|169208597 | 169208597 |
| **17** | 62 | hypothetical protein gi\|34365490 | 34365490 |
| **18** | 45 | Hypothetical protein gi\|51476390 | 51476390 |
| **19** | 37 | Hypothetical protein gi\|51476513 | 51476513 |
| **20** | 37 | hypothetical protein gi\|59006610 | 59006610 |
| **21** | 39 | Hypothetical protein gi\|5911993 | 5911993 |
| **22** | 33 | Hypothetical protein gi\|6453547 | 6453547 |
| **23** | 44 | Hypothetical protein gi\|7018414 | 7018414 |
| **24** | 31 | Hypothetical protein LOC147685 | 22748999 |
| **25** | 53 | Hypothetical protein LOC255798 | 117938326 |
| **26** | 30 | Hypothetical protein LOC286009 | 51095120 |
| **27** | 73 | Hypothetical protein LOC286257 | 39930541 |
| **28** | 43 | Hypothetical protein LOC57821 | 10880975 |
| **29** | 32 | KIAA0115 protein | 473947 |
| **30** | 39 | KIAA0253 protein | 1665773 |
| **31** | 31 | KIAA0285 protein | 20521025 |
| **32** | 31 | KIAA0423 protein | 20521047 |
| **33** | 37 | KIAA0445 protein | 29421172 |
| **34** | 31 | KIAA0624 protein |  |
| **35** | 37 | KIAA0753 protein | 40788352 |
| **36** | 30 | KIAA0828 protein |  |
| **37** | 44 | KIAA1010 protein |  |
| **38** | 31 | KIAA1035 protein | 20521740 |
| **39** | 33 | KIAA1078 protein | 20521756 |
| **40** | 33 | KIAA1454 protein |  |
| **41** | 51 | KIAA1481 protein |  |
| **42** | 71 | KIAA1727 protein | 12697999 |
| **43** | 30 | LOC150763 protein | 40352834 |
| **44** | 69 | LOC644936 protein | 62204157 |
| **45** | 50 | Unamed protein product gi\|22760986 | 22760986 |
| **46** | 37 | Unknown | 10503972 |
| **47** | 30 | Unknown (protein for IMAGE:40134249) | 121934000 |
| **48** | 58 | Unknown protein precursor gi\|393315 | 393315 |
| **49** | 31 | Unnamed protein product gi\|10438150 | 10438150 |
| **50** | 56 | Unnamed protein product gi\|10434265 | 10434265 |
| **51** | 93 | Unnamed protein product gi\|10435945 | 10435945 |
| **52** | 62 | Unnamed protein product gi\|10437832 | 10437832 |
| **53** | 31 | Unnamed protein product gi\|158255342 | 158255342 |
| **54** | 33 | Unnamed protein product gi\|16550641 | 16550641 |
| **55** | 87 | Unnamed protein product gi\|16553735 | 16553735 |
| **56** | 54 | Unnamed protein product gi\|21750376 | 21750376 |
| **57** | 29 | Unnamed protein product gi\|21750626 | 21750626 |
| **58** | 33 | Unnamed protein product gi\|21751916 | 21751916 |
| **59** | 37 | Unnamed protein product gi\|21758400 | 21758400 |
| **60** | 37 | Unnamed protein product gi\|22760302 | 22760302 |
| **61** | 35 | Unnamed protein product gi\|22760716 | 22760716 |
| **62** | 58 | Unnamed protein product gi\|28207867 | 28207867 |
| **63** | 46 | Unnamed protein product gi\|28606 | 28606 |
| **64** | 35 | Unnamed protein product gi\|28872 | 28872 |
| **65** | 61 | Unnamed protein product gi\|29577 | 29577 |
| **66** | 61 | Unnamed protein product gi\|29888 | 29888 |
| **67** | 82 | Unnamed protein product gi\|29900 | 29900 |
| **68** | 66 | Unnamed protein product gi\|31291 | 31291 |
| **69** | 31 | Unnamed protein product gi\|31543 | 31543 |
| **70** | 31 | Unnamed protein product gi\|31714 | 31714 |
| **71** | 61 | Unnamed protein product gi\|31865 | 31865 |
| **72** | 73 | Unnamed protein product gi\|32458 | 32458 |
| **73** | 33 | Unnamed protein product gi\|32488 | 32488 |
| **74** | 36 | Unnamed protein product gi\|34429 | 34429 |
| **75** | 40 | Unnamed protein product gi\|34526394 | 34526394 |
| **76** | 31 | Unnamed protein product gi\|34758 | 34758 |
| **77** | 76 | Unnamed protein product gi\|34999 | 34999 |
| **78** | 71 | Unnamed protein product gi\|35193 | 35193 |
| **79** | 34 | Unnamed protein product gi\|35218 | 35218 |
| **80** | 38 | Unnamed protein product gi\|36535 | 36535 |
| **81** | 85 | Unnamed protein product gi\|37460 | 37460 |
| **82** | 51 | Unnamed protein product gi\|37593 | 37593 |
| **83** | 41 | Unnamed protein product gi\|38267 | 38267 |
| **84** | 30 | Unnamed protein product gi\|7020119 | 7020119 |
| **85** | 63 | Unnamed protein product gi\|7020438 | 7020438 |
| **86** | 43 | Unnamed protein product gi\|7022407 | 7022407 |
| **87** | 33 | Unnamed protein product gi\|7022608 | 7022608 |
| **88** | 38 | Unnamed protein product gi\|7022952 | 7022952 |
| **89** | 40 | Unnamed protein product gi\|7023053 | 7023053 |
| **90** | 68 | Unnamed protein product gi\|7023756 | 7023756 |
| **91** | 36 | Unnamed protein product gi\|929623 | 929623 |
