# Supplementary material for: S100A9 is a Biliary Protein Marker of Disease Activity in Primary Sclerosing Cholangitis
Source: PLoS One. 2012 Jan 11;7(1):e29821. doi: 10.1371/journal.pone.0029821 (PMC3256182; doi:10.1371/journal.pone.0029821)
Supplement: Table S3 — List of bile duct-derived bile proteins identified with a Mascot score <100. (DOCX) [file pone.0029821.s003.docx]

**Supplemental Table 3**

List of bile duct-derived bile proteins identified with a Mascot score < 100.

| **No.** | **Protein name** | **Score** |
| --- | --- | --- |
| 1 | 2,4-dienoyl-CoA reductase | 60 |
| 2 | 2-phosphopyruvate-hydratase alpha-enolase; carbonate dehydratase | 49 |
| 3 | 70 kD alpha-glucosidase | 94 |
| 4 | ABC transporter | 66 |
| 5 | Abhydrolase domain containing 10 | 58 |
| 6 | Abhydrolase domain containing 14B | 35 |
| 7 | Abnormal spindle protein ASP | 38 |
| 8 | Absent in melanoma 2 | 43 |
| 9 | Actin related protein 2/3 complex subunit 2 | 42 |
| 10 | Actin related protein 2/3 complex subunit 4 isoform a | 56 |
| 11 | Actin related protein 2/3 complex subunit 5 | 39 |
| 12 | Actin-like protein | 56 |
| 13 | ADAM metallopeptidase | 39 |
| 14 | Adenylyl cyclase-associated protein | 37 |
| 15 | Adipsin/complement factor D | 35 |
| 16 | ADP-Ribosylation Factor | 54 |
| 17 | ADP-ribosylation factor 6 | 97 |
| 18 | ADP-ribosylation factor domain protein 1 isoform alpha | 32 |
| 19 | ADP-ribosylation factor interacting protein 1 isoform 2 | 44 |
| 20 | Advillin | 37 |
| 21 | AF1 non-allergic IgE heavy chain IGHV4-61 | 42 |
| 22 | AF2 non-allergic IgE heavy chain IGHV3-23 | 85 |
| 23 | AKR1CL2 protein | 31 |
| 24 | Alcohol dehydrogenase 4 (Alcohol dehydrogenase class II pi chain) | 86 |
| 25 | Aldehyde dehydrogenase | 40 |
| 26 | Aldehyde dehydrogenase 1A1 | 56 |
| 27 | Aldehyde dehydrogenase 8A1 | 33 |
| 28 | Aldo-keto reductase family 1, member A1 | 61 |
| 29 | Aldo-keto reductase family 1, member D1 | 56 |
| 30 | Aldolase B | 51 |
| 31 | Alpha 2-plasmin inhibitor | 40 |
| 32 | Alpha-1 type I collagen | 40 |
| 33 | Alpha1-acid glycoprotein | 81 |
| 34 | Alpha-fodrin | 47 |
| 35 | Alpha-tubulin | 30 |
| 36 | Aminopeptidase B | 34 |
| 37 | Annexin A13 | 30 |
| 38 | Annexin A2 isoform 2 | 81 |
| 39 | Annexin A3 | 34 |
| 40 | Annexin V | 39 |
| 41 | Anterior gradient 2 homolog | 63 |
| 42 | Anti-folate binding protein | 42 |
| 43 | Apolipoprotein C-III | 82 |
| 44 | Apolipoprotein E | 96 |
| 45 | Apolipoprotein L-I | 34 |
| 46 | Apurinic/apyrimidinic endonuclease | 57 |
| 47 | Archain | 85 |
| 48 | Arginase (EC 3.5.3.1) | 91 |
| 49 | ARP3 homolog | 84 |
| 50 | AS1 | 44 |
| 51 | Asialoglycoprotein receptor 1 | 40 |
| 52 | Asparaginase-like protein | 42 |
| 53 | Aspartate aminotransferase 1 | 60 |
| 54 | ATAD2 | 96 |
| 55 | ATP synthase, H+ transporting, mitochondrial F1 complex, delta subunit precursor | 75 |
| 56 | ATP-binding cassette, sub-family B (MDR/TAP), member 9 | 39 |
| 57 | Azurocidin | 35 |
| 58 | B cell differentiation factor I | 36 |
| 59 | Bactericidal/permeability-increasing protein-like 2 | 39 |
| 60 | Beta-defensin-1 | 32 |
| 61 | Beta-hexosaminidase | 27 |
| 62 | Beta-trace=23.5 kda glycoprotein {N-terminal} [human, cerebrospinal fluid, Peptide Partial, 19 aa] | 40 |
| 63 | BIGH3 | 52 |
| 64 | Biliverdin-IX beta reductase isozyme I | 46 |
| 65 | Biotinidase precursor (Biotinase) | 61 |
| 66 | Blood plasma glutamate carboxypeptidase precursor; prostate-specific membrane antigen (PSMA) | 65 |
| 67 | C1-inhibitor | 42 |
| 68 | C9 complement protein | 57 |
| 69 | CAGH32 | 31 |
| 70 | Calcium-binding protein 39-like | 21 |
| 71 | Calcyphosine isoform a | 44 |
| 72 | Calreticulin | 63 |
| 73 | Capping protein alpha | 63 |
| 74 | Carbonyl reductase 3 | 92 |
| 75 | Carboxymethylenebutenolidase | 22 |
| 76 | Carboxypeptidase B | 78 |
| 77 | Carboxypeptidase N precursor | 55 |
| 78 | Cartilage intermediate layer protein 2 | 23 |
| 79 | Cartilage oligomeric matrix protein precursor (COMP) | 87 |
| 80 | Cathepsin H | 45 |
| 81 | Cathepsin L1 | 47 |
| 82 | Cathepsin S | 52 |
| 83 | Cationic trypsinogen | 65 |
| 84 | CD59 antigen preproprotein | 33 |
| 85 | CD63 antigen isoform A | 40 |
| 86 | CDS | 30 |
| 87 | CGI-44 | 93 |
| 88 | CHADL | 36 |
| 89 | Chlordecone reductase | 57 |
| 90 | Chymotrypsin | 52 |
| 91 | Chymotrypsinogen B2 precursor [Contains: Chymotrypsin B2 chain A; Chymotrypsin B2 chain B; Chymotrypsin B2 chain C] | 90 |
| 92 | CLA-1 | 47 |
| 93 | Clarin 3 | 57 |
| 94 | class V alcohol dehydrogenase 6 isoform 2 | 67 |
| 95 | Clathrin heavy chain 1 | 50 |
| 96 | C-myc binding protein | 33 |
| 97 | Coagulation factor XII precursor (Hageman factor) (HAF) [Contains: Coagulation factor XIIa heavy chain; Beta-factor XIIa part 1; Beta-factor XIIa part 2; Coagulation factor XIIa light chain] | 82 |
| 98 | Cofilin 1 (non-muscle) | 57 |
| 99 | Cofilin 2 | 44 |
| 100 | Coiled-coil domain-containing protein 13 | 30 |
| 101 | Colipase preproprotein | 69 |
| 102 | Collagen alpha-3(VI) chain precursor | 82 |
| 103 | Complement 8 alpha subunit | 57 |
| 104 | Complement C1q subcomponent subunit B precursor | 74 |
| 105 | Complement C1r activated form | 35 |
| 106 | Complement C1r subcomponent precursor (Complement component 1, r subcomponent) [Contains: Complement C1r subcomponent heavy chain; Complement C1r subcomponent light chain] | 79 |
| 107 | Complement C4B precursor | 78 |
| 108 | Complement component 1, r subcomponent-like precursor | 61 |
| 109 | Complement component C1s=serine protease [human, plasma, Peptide Partial, 15 aa, segment 10 of 11] | 40 |
| 110 | Complement Factor H-related Protein 2 | 71 |
| 111 | Complement protein C7 precursor | 58 |
| 112 | Completement component C6 | 41 |
| 113 | Complex-forming glycoprotein HC | 76 |
| 114 | Creatine kinase-B | 60 |
| 115 | C-type lectin | 34 |
| 116 | Cystatin B | 50 |
| 117 | Cystatin M precursor | 73 |
| 118 | Cytochrome b5 | 40 |
| 119 | Cytochrome c | 42 |
| 120 | Cytokeratin 18 (424 AA) | 36 |
| 121 | Cytoplasmic phosphotyrosyl protein phosphatase | 67 |
| 122 | Cytosolic inorganic pyrophosphatase | 61 |
| 123 | Cytosolic malate dehydrogenase | 39 |
| 124 | DAZ interacting protein | 31 |
| 125 | Cecay-acceleration factor | 52 |
| 126 | Celta-aminolevulinate dehydratase; ALAD | 50 |
| 127 | Dermcidin | 63 |
| 128 | Desmocollin type 2a | 38 |
| 129 | Cesmoglein 2 | 49 |
| 130 | Dihydrolipoyl dehydrogenase, mitochondrial precursor (Dihydrolipoamide dehydrogenase) (Glycine cleavage system L protein) | 79 |
| 131 | Disks large-associated protein 4 | 53 |
| 132 | DJ-1 | 57 |
| 133 | DNA polymerase epsilon catalytic subunit | 33 |
| 134 | DNA polymerase eta | 56 |
| 135 | DNAJ Homolog | 34 |
| 136 | Double stranded RNA activated protein kinase | 34 |
| 137 | Doublesex-mab-3 (DM) domain | 32 |
| 138 | Dynein | 78 |
| 139 | E2N | 98 |
| 140 | E-cadherin | 30 |
| 141 | ELK3 | 37 |
| 142 | Elongation factor 1 | 71 |
| 143 | Elongin A | 40 |
| 144 | Elongin B isoform a | 33 |
| 145 | EMILIN-2 | 41 |
| 146 | Endoplasmic reticulum protein 29 isoform 1 precursor | 36 |
| 147 | Epididymal secretory protein E1 precursor | 61 |
| 148 | Epithelial cell marker protein 1 | 59 |
| 149 | Esterase D | 54 |
| 150 | Eukaryotic initiation factor 4AII | 72 |
| 151 | F11 receptor precursor | 89 |
| 152 | F-actin capping protein beta subunit | 36 |
| 153 | Factor H | 77 |
| 154 | FALL-39 peptide antibiotic | 46 |
| 155 | FAM151A protein | 52 |
| 156 | Fas-ligand associated factor 3 | 36 |
| 157 | F-box only protein 5 | 31 |
| 158 | FHR-1; complement factor H-related Protein 1 | 76 |
| 159 | Fibronectin type III | 46 |
| 160 | Filaggrin | 42 |
| 161 | FIP2 | 41 |
| 162 | FLJ16008 protein | 65 |
| 163 | FLJ37218 | 41 |
| 164 | FOS-like antigen 1 | 36 |
| 165 | Fructose-1,6-bisphosphatase 1 (FBPase 1) (D-fructose-1,6-bisphosphate 1-phosphohydrolase 1) | 44 |
| 166 | Furin | 47 |
| 167 | FUSE binding protein 3 | 42 |
| 168 | G protein beta subunit | 56 |
| 169 | G3a | 36 |
| 170 | Ga subunit | 61 |
| 171 | Gamma-interferon inducible early response polypeptide | 43 |
| 172 | GAPDH-2 like | 96 |
| 173 | GDNF family receptor alpha 1 isoform a preproprotein | 44 |
| 174 | GEF 17 | 35 |
| 175 | Glucosamine (N-acetyl)-6-sulfatase precursor | 69 |
| 176 | Glutamate--ammonia ligase | 42 |
| 177 | Glutamate--cysteine ligase | 68 |
| 178 | Glutamine-fructose-6-phosphate transaminase 2 | 32 |
| 179 | Glutathione transferase | 55 |
| 180 | Gluthione S-transferase subunit 1 (GST,EC 2.5.1.18) | 74 |
| 181 | Glyoxylate reductase/hydroxypyruvate reductase | 31 |
| 182 | Glypican 6 precursor | 61 |
| 183 | Golgi membrane protein GP73 | 78 |
| 184 | GPR115 | 82 |
| 185 | Grancalcin | 31 |
| 186 | Growth arrest-specific 8 | 30 |
| 187 | GRP78 | 35 |
| 188 | GTP binding protein | 41 |
| 189 | Guanidinoacetate N-methyltransferase | 90 |
| 190 | Guanine nucleotide binding protein (G protein), beta polypeptide 2-like 1 | 83 |
| 191 | Heat shock protein 27 | 96 |
| 192 | Heat shock-induced protein | 58 |
| 193 | Heat-responsive protein 12 | 90 |
| 194 | Heme binding protein 2 | 33 |
| 195 | Heparin cofactor II precursor | 42 |
| 196 | Heparin cofactor li | 57 |
| 197 | Hepatitis delta antigen interacting protein A | 30 |
| 198 | HGF receptor | 58 |
| 199 | Histone H3 | 77 |
| 200 | Horf6 | 83 |
| 201 | HSPC135 | 36 |
| 202 | HSPC336 | 58 |
| 203 | Human elongation factor-1-delta | 35 |
| 204 | Hydroxypyruvate isomerase homolog | 42 |
| 205 | Hydroxysteroid (17-beta) dehydrogenase 4 | 41 |
| 206 | ICAM-2 preprotein (AA -21 to 254) | 35 |
| 207 | InsP3 receptor | 37 |
| 208 | InsP3R2 | 30 |
| 209 | Insulin-like growth factor binding protein 7 | 84 |
| 210 | Integrin beta-1 precursor (Fibronectin receptor subunit beta) (Integrin VLA-4 subunit beta) (CD29 antigen) | 80 |
| 211 | Integrin beta-5 subunit precursor | 35 |
| 212 | Interleukin 1 receptor accessory protein isoform 1 | 40 |
| 213 | interleukin-1 receptor accessory protein | 60 |
| 214 | Intestinal trefoil factor | 69 |
| 215 | Isochorismatase domain containing 2 | 35 |
| 216 | Junction adhesion molecule | 40 |
| 217 | Junctophilin 1 | 32 |
| 218 | Katanin p60 subunit A-like 2 | 32 |
| 219 | Keratin 1 | 31 |
| 220 | Keratin 9 | 50 |
| 221 | Keratin, type I cytoskeletal 12 | 31 |
| 222 | Keratin, type I cytoskeletal 16 | 69 |
| 223 | Keratin, type I cytoskeletal 18 | 50 |
| 224 | Keratin, type I cytoskeletal 28 | 45 |
| 225 | Keratin, type II cytoskeletal 1 | 55 |
| 226 | Keratin, type II cytoskeletal 5 | 45 |
| 227 | Keratin, type II cytoskeletal 72 | 21 |
| 228 | Ki nuclear autoantigen | 67 |
| 229 | KIF4 | 34 |
| 230 | Kinase suppressor of Ras-2 | 41 |
| 231 | Kininogen 1 isoform 2 | 60 |
| 232 | Kininogen light chain | 68 |
| 233 | L apoferritin | 35 |
| 234 | L21 | 32 |
| 235 | Llactate dehydrogenase-C | 31 |
| 236 | Latexin | 46 |
| 237 | LEKTI | 43 |
| 238 | Leptin receptor | 37 |
| 239 | Leukocyte adhesion glycoprotein precursor | 77 |
| 240 | leukocyte adhesion protein beta-subunit precursor | 76 |
| 241 | Lipocalin 1 | 61 |
| 242 | LPS-binding protein, LBP=lipopolysaccharide-binding protein [human, liver, Peptide, 481 aa] | 36 |
| 243 | Lymphatic vessel endothelial hyaluronan receptor 1 | 51 |
| 244 | Lysosomal membrane glycoprotein-2 | 47 |
| 245 | Lysosome-associated membrane glycoprotein 1 precursor (LAMP-1) (CD107 antigen-like family member A) (CD107a antigen) | 45 |
| 246 | MAC25 | 48 |
| 247 | Macrophage migration inhibitory factor | 95 |
| 248 | Manganese superoxide dismutase (MnSOD) | 47 |
| 249 | MEGF7 | 43 |
| 250 | Melanotransferrin precursor (Melanoma-associated antigen p97) (CD228 antigen) | 43 |
| 251 | Mitochondrial short-chain enoyl-CoA hydratase | 42 |
| 252 | Moesin | 30 |
| 253 | Mono-ADP-ribosyltransferase | 52 |
| 254 | MOP-4 | 32 |
| 255 | Mucin glycoprotein | 45 |
| 256 | Mucin-5AC precursor (Mucin-5 subtype AC, tracheobronchial) (Tracheobronchial mucin) (TBM) (Major airway glycoprotein) (Gastric mucin) (Lewis B blood group antigen) (LeB) | 74 |
| 257 | Multidrug resistance protein | 63 |
| 258 | Myelin basic protein specific T-cell receptor V | 40 |
| 259 | Myosin I homologue | 31 |
| 260 | Na+ K+ ATPase alpha subunit | 63 |
| 261 | NAD(P)H menadione oxidoreductase 1 | 61 |
| 262 | NADH dehydrogenase subunit 2 | 34 |
| 263 | NADPH binding protein | 53 |
| 264 | Nephrocystin 3 splice variant | 33 |
| 265 | Neuroleukin | 80 |
| 266 | Neutrophil elastase precursor | 51 |
| 267 | Neutrophil granule peptide HP1 | 52 |
| 268 | NGAL | 87 |
| 269 | NIMA-related kinase 5 | 82 |
| 270 | Nitrilase family, member 2 | 31 |
| 271 | Non-receptor Tyr kinase | 34 |
| 272 | NSAP1 | 43 |
| 273 | Nuclear distribution gene E homolog 1 | 52 |
| 274 | Nuclear RNA helicase | 94 |
| 275 | Nucleoside triphosphate diphosphohydrolase-8 | 39 |
| 276 | NVL | 43 |
| 277 | Olfactory receptor | 70 |
| 278 | OLFM4 protein | 68 |
| 279 | Ornithine carbamoyltransferase | 24 |
| 280 | Osteoblast specific factor 2 | 41 |
| 281 | Outer dense fiber protein 2/2 | 37 |
| 282 | P107 | 31 |
| 283 | P18SRP protein | 48 |
| 284 | P20 | 34 |
| 285 | P64 CLCP | 61 |
| 286 | Pancreatic GP2 | 54 |
| 287 | Pancreatic lipase-related protein 2 precursor | 54 |
| 288 | PAP-inositol-1,4-phosphatase | 81 |
| 289 | Paraoxonase 2 | 72 |
| 290 | Paraoxonase-3 | 43 |
| 291 | Parkinson disease protein 7 | 80 |
| 292 | PDE4D5 | 81 |
| 293 | Peptidoglycan recognition protein 1 | 60 |
| 294 | Peptidyl-prolyl cis-trans isomerase B precursor (PPIase) (Rotamase) (Cyclophilin B) (S-cyclophilin) (SCYLP) (CYP-S1) | 89 |
| 295 | Peptidylprolyl isomerase | 36 |
| 296 | Peroxiredoxin 3 | 32 |
| 297 | Peroxiredoxin-4 | 60 |
| 298 | Peroxisomal Delta3, Delta2 Enoyl Coa Isomerase | 34 |
| 299 | P-glycoprotein | 44 |
| 300 | PH and SEC7 domain-containing protein 2 | 28 |
| 301 | Phenylalanine hydroxylase-stimulating protein, pterin-4 alpha-carbinolamine dehydratase, PHS, PCD [human, liver, Peptide, 103 aa] | 70 |
| 302 | phospholipid transfer protein isoform a precursor | 41 |
| 303 | Phosphomannomutase 2 | 23 |
| 304 | Phosphorylase b kinase | 24 |
| 305 | PHP | 90 |
| 306 | PHP; putative heart protein | 66 |
| 307 | PI3K | 30 |
| 308 | Pig3 | 40 |
| 309 | Pigment epithelial-differentiating factor | 86 |
| 310 | Plasma glutathione peroxidase | 75 |
| 311 | Plasma kallikrein precursor (Plasma prekallikrein) (Kininogenin) (Fletcher factor) [Contains: Plasma kallikrein heavy chain; Plasma kallikrein light chain] | 92 |
| 312 | Platelet-activating factor acetylhydrolase IB | 39 |
| 313 | Platelet-activating factor acetylhydrolase, isoform Ib, beta subunit 30kDa | 55 |
| 314 | Platelet-activating factor acetylhydrolase, isoform Ib, gamma subunit 29kDa | 48 |
| 315 | Poliovirus receptor | 77 |
| 316 | Poly(A) binding protein interacting protein 1 | 35 |
| 317 | Polyubiquitin | 96 |
| 318 | Porin 31HM | 43 |
| 319 | Potassium voltage-gated channel, subfamily H, member 5 | 44 |
| 320 | Precursor polypeptide (AA -21 to 494) | 84 |
| 321 | PREDICTED: hypothetical protein | 51 |
| 322 | PREDICTED: similar to lipoprotein Lp(a) | 61 |
| 323 | PREDICTED: similar to Putative ubiquitin-conjugating enzyme E2 D3-like protein | 37 |
| 324 | Pregnancy zone protein | 44 |
| 325 | Prepro-C3b/C4B inactivator | 99 |
| 326 | Pre-pro-megakaryocyte potentiating factor | 59 |
| 327 | Prion protein | 37 |
| 328 | Profilin 1 | 43 |
| 329 | Progesterone receptor membrane component 2 | 53 |
| 330 | Programmed cell death 6 | 35 |
| 331 | Prohibitin 2 | 23 |
| 332 | Prolidase | 51 |
| 333 | Prominin 1 | 57 |
| 334 | Prostasin preproprotein | 80 |
| 335 | Prostate-specific membrane antigen | 48 |
| 336 | Protease serine 1 | 83 |
| 337 | Proteasome (prosome, macropain) subunit | 93 |
| 338 | proteasome (prosome, macropain) subunit, alpha type 6 [Rattus norvegicus] | 36 |
| 339 | Proteasome activator subunit 1 isoform 1 | 51 |
| 340 | Proteasome alpha 3 subunit isoform 1 | 33 |
| 341 | Proteasome subint beta type-4 | 68 |
| 342 | Proteasome subint beta type-7 | 93 |
| 343 | Proteasome subunit LMP7 | 87 |
| 344 | Protein arginine N-methyltransferase 1 | 55 |
| 345 | protein disulfide isomerase-associated 4 | 57 |
| 346 | protein disulfide isomerase-related protein 5 | 67 |
| 347 | Protein-kinase, interferon-inducible double stranded RNA dependent inhibitor, repressor of (P58 repressor) | 37 |
| 348 | Protein-serine/threonine kinase | 82 |
| 349 | Purine nucleoside phosphorylase | 65 |
| 350 | Pyruvate kinase | 46 |
| 351 | Quiescin Q6 sulfhydryl oxidase 1 isoform a | 59 |
| 352 | Quinoid dihydropteridine reductase | 79 |
| 353 | R33729_1 | 54 |
| 354 | Rab11-family interacting protein 3 | 43 |
| 355 | RAB27B, member RAS oncogene family | 41 |
| 356 | RAB33B, member RAS oncogene family | 81 |
| 357 | RAB4B protein | 83 |
| 358 | Rab5c-like protein, similar to Canis familiaris Rab5c protein, PIR Accession Number S38625 | 51 |
| 359 | RAB7B | 29 |
| 360 | Rab-related GTP-binding protein | 81 |
| 361 | RAD52 | 32 |
| 362 | RAD54-like protein | 34 |
| 363 | Regenerating protein (reg) | 53 |
| 364 | Regulator of G-protein signaling 4 isoform 2 | 33 |
| 365 | Reticulocalbin 1 | 30 |
| 366 | Reticulon 4 receptor-like 2 | 50 |
| 367 | Retinol binding protein 2, cellular | 63 |
| 368 | Rheumatoid factor | 43 |
| 369 | Rho GDP dissociation inhibitor (GDI) alpha | 54 |
| 370 | RIB43A domain with coiled-coils 1 isoform 1 | 31 |
| 371 | Ribose 5-phosphate isomerase A | 49 |
| 372 | Ribosomal protein L22 | 36 |
| 373 | ribosomal protein L7 | 42 |
| 374 | Ribosomal protein S11 | 33 |
| 375 | Ribosomal protein S14 | 58 |
| 376 | Ribosomal protein S19 | 66 |
| 377 | Ribosomal protein S5 | 41 |
| 378 | RNA helicase A | 43 |
| 379 | rotavirus-specific intestinal-homing antibody light chain variable region | 90 |
| 380 | Rough Deal homolog, centromere/kinetochore protein | 32 |
| 381 | Ryanodine receptor 2 (RYR-2) (RyR2) (hRYR-2) (Cardiac muscle-type ryanodine receptor) (Cardiac muscle ryanodine receptor-calcium release channel) | 32 |
| 382 | S plasma protein | 96 |
| 383 | S100 calcium binding protein A11 | 75 |
| 384 | S3 ribosomal protein | 49 |
| 385 | SAR1a gene homolog 2 | 29 |
| 386 | SDCCAG1 | 52 |
| 387 | SEC13 protein | 46 |
| 388 | Secretory leukocyte peptidase inhibitor precursor | 51 |
| 389 | Selenophosphate synthetase 2 | 31 |
| 390 | Semaphorin F homolog | 45 |
| 391 | SGP28 protein | 31 |
| 392 | SH3 domain binding glutamic acid-rich protein like 3 | 74 |
| 393 | SH3-domain GRB2-like endophilin B2 | 37 |
| 394 | SIGLECP16 protein | 37 |
| 395 | Signal-regulatory protein alpha precursor | 63 |
| 396 | Similar to NADH dehydrogenase [ubiquinone] iron-sulfur protein 5 (NADH-ubiquinone oxidoreductase | 36 |
| 397 | Similar to S-adenosylhomocysteine hydrolase | 33 |
| 398 | Similar to sialic acid binding Ig-like lectin 11 | 55 |
| 399 | SIRP-beta1 | 46 |
| 400 | SMAF1 | 49 |
| 401 | Small G protein | 57 |
| 402 | Smooth muscle cell associated protein-1 isoform 2 | 34 |
| 403 | Smooth muscle myosin alkali light chain | 88 |
| 404 | Sodium channel protein type 11 | 28 |
| 405 | Solute carrier family 2 | 35 |
| 406 | Solute carrier family 25 (mitochondrial carrier; adenine nucleotide translocator), member 6 | 32 |
| 407 | Solute carrier family 9 (sodium/hydrogen exchanger), isoform 3 regulator 1 | 43 |
| 408 | Sop2p-like protein | 34 |
| 409 | Spasmolytic polypeptide | 32 |
| 410 | Spermine Synthase | 41 |
| 411 | Stathmin 1 | 46 |
| 412 | Structural maintenance of chromosomes | 39 |
| 413 | SWI/SNF | 36 |
| 414 | TB3-1 | 36 |
| 415 | TBX3 | 36 |
| 416 | T-cell receptor V beta-specific IgM kappa autoantibody light chain variable region | 31 |
| 417 | Tenascin R (restrictin, janusin) | 35 |
| 418 | Tetranectin precursor (TN) (C-type lectin domain family 3 member B) (Plasminogen kringle 4-binding protein) | 52 |
| 419 | Thioredoxin peroxidase PMP20 | 73 |
| 420 | Thrombin inhibitor | 47 |
| 421 | Thrombospondin 3 precursor | 39 |
| 422 | Thyroid receptor interactor | 60 |
| 423 | Thyroxine-binding globulin precursor (T4-binding globulin) (Serpin A7) | 50 |
| 424 | TIF1gamma | 34 |
| 425 | Tissue inhibitor of metalloproteinases, Type-2 | 40 |
| 426 | TM2-CEA precursor | 50 |
| 427 | TM3-CEA protein | 83 |
| 428 | Transaldolase 1 | 52 |
| 429 | Transformation upregulated nuclear protein | 35 |
| 430 | Transgelin 2 | 97 |
| 431 | Translation initiation factor 6 | 31 |
| 432 | Transmembrane 4 superfamily member 1 | 46 |
| 433 | Transmembrane protein 109 | 33 |
| 434 | Transmembrane protein 198 | 74 |
| 435 | Transmembrane protein 33 | 31 |
| 436 | Transmembrane secretory component | 38 |
| 437 | Trehalase precursor (Alpha,alpha-trehalase) (Alpha,alpha-trehalose glucohydrolase) | 76 |
| 438 | Trinucleotide repeat-containing gene 6A protein (CAG repeat protein 26) (Glycine-tryptophan protein of 182 kDa) (GW182 autoantigen) (Protein GW1) (EMSY interactor protein) | 32 |
| 439 | Triosephosphate isomerase (TIM) (Triose-phosphate isomerase) | 68 |
| 440 | Tripartite motif-containing 6 isoform 2 | 43 |
| 441 | Tropomyosin 4 | 79 |
| 442 | Tubulin | 47 |
| 443 | Tumor necrosis factor (ligand) superfamily, member 13b | 42 |
| 444 | Tumor necrosis factor type 1 receptor associated protein TRAP-1 | 74 |
| 445 | Tumor protein, translationally-controlled 1 | 38 |
| 446 | Tyrosine 3-monooxygenase/tryptophan 5-monooxygenase activation protein, gamma polypeptide [Rattus norvegicus] | 85 |
| 447 | Tyrosine kinase activator protein 1 (TKA-1) | 48 |
| 448 | Ubiquilin 4, isoform CRA_b | 41 |
| 449 | Ubiquitin | 96 |
| 450 | Ubiquitin activating enzyme E1 | 61 |
| 451 | Ubiquitin conjugating enzyme | 37 |
| 452 | Ubiquitin-conjugating enzyme E2N | 70 |
| 453 | UDP-galactose-4-epimerase | 29 |
| 454 | ULIP | 30 |
| 455 | Vacuolar protein sorting 29 isoform 1 | 41 |
| 456 | Valosin-containing protein | 44 |
| 457 | vascular cell adhesion molecule 1 isoform a precursor | 73 |
| 458 | Vimetin | 59 |
| 459 | Vinculin isoform VCL | 41 |
| 460 | VNN1 | 98 |
| 461 | Voltage-dependent anion channel 3 | 43 |
| 462 | WD repeat-containing protein 87 | 45 |
| 463 | WDR1 protein | 33 |
| 464 | X-prolyl aminopeptidase (aminopeptidase P) 3, putative | 35 |
| 465 | YPT3 | 37 |
| 466 | ZFP 42 homolog | 31 |
| 467 | Zinc finger CCHC-type and RNA binding motif 1 | 43 |
