# Supplementary material for: S100A9 is a Biliary Protein Marker of Disease Activity in Primary Sclerosing Cholangitis
Source: PLoS One. 2012 Jan 11;7(1):e29821. doi: 10.1371/journal.pone.0029821 (PMC3256182; doi:10.1371/journal.pone.0029821)
Supplement: Table S4 — List of pathways to which duct-derived bile proteins belong. (DOCX) [file pone.0029821.s004.docx]

**Supplemental Table 4**

List of pathways to which bile duct-derived proteins belong.

| Pathway | Number of proteins |
| --- | --- |
| Acute Phase Response Signaling | 21 |
| Digestion and Absorption | 17 |
| Signal transduction | 17 |
| Inflammatory and Stress Response | 15 |
| Cytoskeletal Signaling | 14 |
| Extracellular Matrix and Adhesion Molecules | 14 |
| Complement System | 13 |
| Glycolysis/Gluconeogenesis | 13 |
| Innate Immune Response | 12 |
| Adaptive Immune Response | 11 |
| Endocytosis | 10 |
| Protein processing in endoplasmic reticulum | 10 |
| Fatty Acid Metabolism | 9 |
| Purine/Pyrimidine Metabolism | 9 |
| Transport | 9 |
| Glutathione metabolism | 8 |
| Translational Control | 8 |
| Coagulation Cascades | 7 |
| Drug Metabolism | 7 |
| Protein Ubiquitination Pathway | 6 |
| Steroid hormone biosynthesis | 5 |
| Apoptosis / Autophagy | 4 |
| Carbohydrate metabolism | 4 |
| Chromatin Regulation / Epigenetics | 4 |
| Pentose Phosphate Pathway | 4 |
| Prostaglaninde biosynthesis | 4 |
| Arachidonic acid metabolism | 3 |
| Communication between Innate and Adaptive Immune Cells | 3 |
| DNA Damage Signaling Pathway | 3 |
| Starch and Sucrose Metabolism | 3 |
| Antigen processing and presentation | 2 |
| B Cell Development | 2 |
| Cell Cycle Control | 2 |
| G-Protein Coupled Receptor Signaling | 2 |
| Phosphatidylinositol signaling system | 2 |
| Folate biosynthesis | 1 |
| Hexosamine pathway | 1 |
| Retinol metabolism | 1 |
| Sphingolipid metabolism | 1 |
| Unknown | 19 |
